# Metabolomics analysis of poly(l-lactic acid) nanofibers' performance on PC12 cell differentiation

**DOI:** 10.1093/rb/rbab031

**Published:** 2021-06-21

**Authors:** Xiaoman Su, Yan Huang, Rong Chen, Yiwen Zhang, Meichen He, Xiaoying Lü

**Affiliations:** 1 State Key Laboratory of Bioelectronics, School of Biological Science and Medical Engineering, Southeast University, 2# Si Pailou, Nanjing 210096, China; 2 Department of Research, SQ Medical Device Co., Ltd, 17# Xinghuo Road, Nanjing 211500, China; 3 Co-innovation Center of Neuroregeneration, Nantong University, Nantong, Jiangsu Province 226019, China

**Keywords:** nanofibers, metabolomics and pathway analysis, PC12 cell differentiation, molecular mechanism

## Abstract

The aim of this article is to reveal the influence of aligned/random poly(l-lactic acid) (PLLA) nanofibers on PC12 cell differentiation from the perspective of metabolic level. First, three materials—PLLA aligned nanofibers (PLLA AF), PLLA random nanofibers (PLLA RF) and PLLA films (control)—were prepared by electrospinning and spin coating. Their surface morphologies were characterized. Subsequently, the cell viability, cell morphology and neurite length of PC12 cells on the surface of the three materials were evaluated, indicating more neurites in the PLLA RF groups but the longer average neurite length in the PLLA AF groups. Next, the metabolite profiles of PC12 cells cultured on the surface of the three nanofibers after 12 h, 24 h and 36 h showed that, compared with the control, 51, 48 and 31 types of differential metabolites were detected at the three time points among the AF groups, respectively; and 56, 45 and 41 types among the RF groups, respectively. Furthermore, the bioinformatics analysis of differential metabolites identified two pathways and three metabolites critical to PC12 cell differentiation influenced by the nanofibers. In addition, the verification experiment on critical metabolites and metabolic pathways were performed. The integrative analysis combining cytology, metabolomics and bioinformatics approaches revealed that though both PLLA AF and RF were capable of stimulating the synthesis of neurotransmitters, the PLLA AF were more beneficial for PC12 cell differentiation, whereas the PLLA RF were less effective.

## Introduction

Poly(l-lactic acid) (PLLA) nanofibers have become a widely used biomaterial in neural tissue engineering [1]. PLLA by itself has good biocompatibility, biodegradability, physical and chemical properties and mechanical strength. PLLA nanofibers provide large contact surface between cells and fibers due to fibers’ high specific surface area; structurally, they resemble the extracellular matrix and thereby provide appropriate contact guidance that facilitates neuronal differentiation and synaptic growth. In addition, the physical properties (such as diameter, density and fiber alignment) of nanofibers do influence cellular behavior on the surface. And the PLLA aligned nanofibers (PLLA AF) promote better contact guidance to support neuronal differentiation and growth than the PLLA random nanofibers (PLLA RF).

A number of studies have evaluated the impact of PLLA AF and PLLA RF on axon growth. Yang *et al*. [2] observed that the direction of axonal growth coincided with that of the aligned nanofibers. Corey *et al*. [1] found that the neurites from dorsal root ganglia explants grew to follow the nanofibers upon contact with the aligned nanofibers, and also noticed that the neurites on the highly aligned nanofibers were 20% longer than those on the random nanofibers. Most of the studies so far focused on the orientation and length of neurite growth, and several gene and protein expressions. However, the molecular mechanisms behind the directional effects of nanofibers on neuronal differentiation yet remain to be extensively explored. Our team has previously investigated the mechanisms of PLLA AF influencing PC12 cell differentiation on the levels of mRNA, microRNA [3, 4] and protein [5].

Following the ideas of genomics and proteomics in this field [6–10], metabolomics research recently has become another powerful method to characterize the interactions between biomaterials and cells. Genomics informs what may happen; proteomics explains what makes it happen; and metabolomics confirms what is happening or has happened [11]. Therefore, many researchers have approached metabolomics technology to study how biomaterials influence cellular functions [12–16]. McNamara *et al.* [13] investigated the mesenchymal stem cell (MSC) responses to titanium nanopillared substrata, and detected the enrichment of certain metabolites that were likely to be engaged in inducing MSC differentiation, such as lipoate, sphingosine and various amino acids. Their work also demonstrated that the nanoscale surfaces sustained stem cell growth and triggered stem cell function [14]. Amer *et al.* analyzed the influence of varying topographical features of poly(d,l-lactic acid) (PLA) microparticles on primary human mesenchymal stem cell attachment, proliferation and markers of osteogenesis. Forty-one metabolites, including stearic acid, oleic acid, etc., were significantly higher in culture media of hMSCs on dimpled PLA compared with smooth microparticle. Key pathways, such as aminoacyl tRNA biosynthesis, amino acid metabolism and energy-based pathways, were significantly up-regulated overall in hMSCs cultured on dimpled PLA microparticles [15]. Chatterjee *et al.* studied the impacts of pristine and functionalized MWCNT on HepG2 and BEAS-2B cells. In general, succinate (in OH, COOH treated cells), pyruvate (in OH treatment), choline (pristine treatment) accumulation in MWCNT exposed HepG2 cells, while betaine, gluathione, o-phosphocholine and taurine depletion in MWCNT treated BEAS-2B cells. Moreover, each type of MWCNT evoke different up/down regulated metabolomic pathways which were further distinct in HepG2 and BEAS-2B cells [16]. However, no research has been reported on the influence of nanofibers on cell differentiation by applying metabolomics approach.

The aim of this article is to investigate the molecular mechanisms of PLLA AF and RF nanofibers on PC12 cell differentiation from the perspective of metabolic level. First, we compared the impacts of PLLA AF, PLLA RF and PLLA films (control) on the cell viability, cell morphology and neurite length. Next, we used metabolomics method to identify differential metabolites on the three materials and performed pathway analysis. Then, we conducted verification experiments for the differential metabolites and metabolic pathways critical to PC12 cell differentiation induced by the nanofibers. Furthermore, we discussed the metebolic mechanisms of PC12 cell differentiation influenced by PLLA nanofibers based on an integrative analysis of cytology, metabolomics, bioinformatics and verification experiments.

## Materials and methods

### Preparation and characterization of PLLA nanofibers

The PLLA (MW = 500 kDa, OH-PLLA-COOR, Jinan Daigang Biomaterial Co., Ltd, China) was dissolved in 1,2-dichloroethane/N, N-Dimethylformamide (v:v = 7:3) to prepare the 1 wt% solution. The 1 wt% solution was used to prepare PLLA films by spin coating on the surface of polystyrene discs (diameter = 8.5 cm), circular glass substrates (diameter = 9 mm), and rectangular glass substrates (1 cm*1 cm), respectively. The PLLA AF and RF were prepared by electrospinning [3] and wrapped onto polystyrene discs or glass substrates. The surface morphologies were characterized by Ultra Plus scanning electron microscope (SEM) (Zeiss, Germany). The average diameters of nanofibers were calculated among 70 randomly selected AF and RF from 5 images of 3 samples via Nano Measurer.

The PLLA substrates were sterilized for 2 h each side by UV lamp before biological experiments. Then the three substrates were soaked in the 0.1 mg/mL poly-l-lysine solution and culture medium for PC12 cells for 4 h, subsequently.

### PC12 cell culture

The undifferentiated PC12 cells (obtained from the Cell Bank at the Chinese Academy of Sciences, Shanghai, China) were cultured and maintained for 48 h in the differentiation medium (95% high-glucose DMEM, Gibco, USA) supplemented with 1% horse serum (HyClone, USA), 3% fetal bovine serum (Sijiqing, China), 1% penicillin-streptomycin (HyClone, USA) and 50 ng/mL nerve growth factor (R&D Systems, USA).

### Cell viability assay (cell counting kit-8 assay, CCK8 assay)

The three rectangular PLLA substrates (1 cm*1 cm) were placed in the 24-well plates. The PC12 cells were seeded with a cell density of 2*10^4^ cells/ml in each well, and cultured in the differentiation medium for 12, 24 and 36 h, respectively. The negative control cells were cultured in the differentiation medium alone; the positive control cells were cultured in the medium plus 0.7% acrylamide solution without any biomaterial. At each time point, the culture medium was removed by vacuum aspiration, and the cells were rinsed twice with phosphate buffer saline (PBS). The differentiation medium plus 1 ml 10% CCK8 solution was added to each well. Cells were incubated for another 3 h. Then, 150 μl of culture was collected from each well into a 96-well plate; and the absorbance at 450 nm was measured on a Multiskan Go spectrophotometer (Thermo Fisher, USA). The cell survival rate was calculated.

### Observation of cell morphology by scanning electron microscopy (SEM)

Cells were cultured for 12, 24 and 36 h, according to the culture procedures described in Section ‘Cell viability assay (Cell Counting Kit-8 assay, CCK8 assay)’. At each time point, the culture medium was removed by vacuum aspiration, and the cells were rinsed twice with PBS. Then, the cells were fixed at 4°C for 2 h using 2.5% glutaraldehyde. The samples were dehydrated with graded ethanol series, starting with 25%, 50%, 75%, 80%, and 95% ethanol, each for 15 min. The gold particles were added after the samples dried. The PC12 cell morphology on the surface of the substrates was observed via a scanning electron microscope.

### Characterization of neurites

Similarly, as described in Section ‘Cell viability assay (Cell Counting Kit-8 assay, CCK8 assay)’, cells were cultured on the circular glass substrates (diameter = 9 mm) for 12, 24 and 36 h, respectively. At each time point, the culture medium was removed by vacuum aspiration, and the cells were rinsed twice with PBS. Then the cells were successively incubated in the 4% paraformaldehyde solution at room temperature for 15 min, in the 0.1% Triton X-100 solution for 10 min and in the 1% bovine serum albumin for 1 h. The cells were stained with 5 μg/ml fluorescein-phalloidin solution (MilliporeSigma, USA) for 30 min and with DAPI solution (MilliporeSigma, USA) for 4 min. The fluorescent imaging was performed on a high content screening (HCS)-high content analysis (HCA) platform (ArrayScan XTI, Thermo Fisher Scientific, USA). The average number of neurites and the average neurite length were calculated.

### Analysis of gene expression—quantitative real-time PCR (qRT-PCR)

The 1 ml PC12 cell suspension with a cell density of 5*10^5^ cells/ml was added on the three PLLA substrates (diameter = 8.5 cm). Cells were cultured for 12 and 36 h. At each time point, cells were collected; the total RNA in the cells was purified and reverse-transcribed to cDNA. The fluorescence-based quantitative PCR was used to measure the expression of a neuronal differentiation marker gene, *GAP43*, as well as the expression of four other genes, *Pafah*1*b*1, *Daam*1, *Slit*2 and *Ascl*1, which play critical roles in such important physical functions as axon outgrowth and nervous system development. *GAPDH* was set as loading control [3].

### Metabolomics experiment

The PC12 cells during neuronal differentiation were seeded with a cell density of 7500 cells/cm^2^ on the PLLA films (control, diameter = 8.5 cm), the PLLA AF substrates (AF groups, diameter = 8.5 cm) and the PLLA RF substrates (RF groups, diameter = 8.5 cm) [3]. Cells were cultured in the differentiation medium for 12, 24 and 36 h, respectively. At each time point, cells were collected; the total intracellular metabolites were extracted. Separation, quantitation and identification of the total metabolites were performed on an Agilent-1290 Infinity Liquid Chromatography system coupled to an Agilent-6530 Accurate-Mass Quadrupole Time-of-Flight/Mass Spectrometer (Agilent, USA) at Shanghai Sensichip Hightech, China. The metabolite profiles were achieved in both positive and negative ion modes. The experiments were replicated five times for all nine groups (three substrates in combination with three incubation times).

The unsupervised principal component analysis (PCA) was conducted by the Simca-P to evaluate the differences within and between groups. The differential metabolites were then determined by the variable importance in the projection (VIP) value greater than 1.0 in the partial least squares discriminant analysis (PLS-DA) and the *P* values of the *t*-test (*P *<* *0.05). The fold changes of metabolites were calculated using base 2 to calculate the log ratio of experiment group/control group; the differential metabolites were determined as significant using *P *<* *0.05. Metabolites with a fold change greater than 0 were considered as up-regulated but less than 0 as down-regulated.

### Bioinformatics analysis for metabolomics results

#### Heat map analysis

The heat map of the differential metabolites in the AF and RF groups was generated using Origin to visualize the variation among samples. The differential metabolites in the AF and RF groups were screened at all three time points.

#### Analysis of metabolic pathways

##### Analysis of metabolic pathways relevant to differential metabolites at three time points

The metabolic pathway analysis was performed by the online MetaboAnalyst (https://www.metaboanalyst.ca/) for the differential metabolites at the three time points in the AF and RF groups. The metabolic pathways relevant to differential metabolites were identified with the pathway impact greater than 0.2 or the negative log_10_ of *P* values greater than 10.

##### Identification of important metabolic pathways for neuronal differentiation

Based on the analysis in Section ‘Analysis of metabolic pathways relevant to differential metabolites at three time points’, the metabolic pathways important to neuronal differentiation were discussed according to literature.

##### Identification of metabolic pathways and metabolites critical to PC12 cell differentiation influenced by PLLA nanofibers

The critical metabolic pathways were required to match all the following criteria: (i) identification of affected differential metabolites at all three time points; (ii) involvement in neuronal differentiation; (iii) minimum pathway impact of 0.6; and (iv) inclusion of at least one metabolite which showed same pattern of differential change at all three time points.

#### Verification of concentration of metabolites and functions of critical metabolic pathways

##### Verification of the concentration of critical metabolites

The concentration of the two critical metabolites, phenylalanine and tyrosine, was performed on an ultra-high pressure liquid chromatography/mass spectrometry (LC-MS) with limit of quantitation platform (Thermo Fisher Scientific, USA) and an Agilent 7890B/5977A Series Gas Chromatograph/Mass Selective Detector (Agilent, USA) at Shanghai Omicsspace Biotech Co., Ltd, China.

##### Verification of the functions of critical metabolic pathway—measurement of Ca^2+^ concentration in PC12 cells

The intracellular calcium ion (Ca^2+^) concentration was measured by a Fluo-3 AM Calcium Indicator (S1056, Beyotime Biotechnology Co., Ltd, China). The 1 ml PC12 cell suspension with a cell density of 5*10^5^ cells/mL was added on the three PLLA substrates. Cells were cultured for 12, 24 and 36 h. At each time point, cells were lysed via trypsin and collected by centrifugation. The 1 ml PBS solution was added to suspend the cells; the Fluo-3 AM solution was added to the concentration of 8 μmol/l. Cells were incubated for 1 h. Then the medium was aspirated after centrifugation. The cells were rinsed twice with PBS and were resuspended with 1 ml PBS. The average Ca^2+^-dependent fluorescence intensity was measured with an excitation wavelength of 488 nm on a FACSCalibur (BD, USA).

### Statistical analysis

All presented data were presented as mean ± standard deviation (mean ± SD), unless stated to use Student’s *t*-test. The level of statistically significance was defined as *P *<* *0.05 and highly statistical significance as *P *<* *0.01. The experiments were replicated at least three times.

## Results and discussion

### Morphology of PLLA substrates

**Figure 1.**
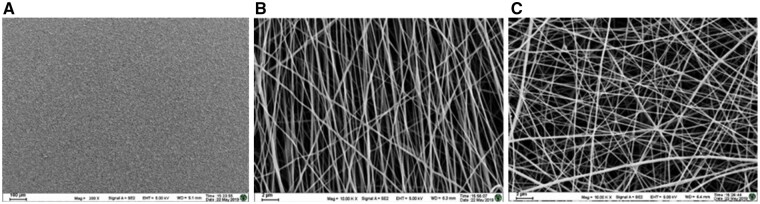
The SEM images of the three PLLA substrates. (**A**) The PLLA films, (**B**) the PLLA AF and (**C**) the PLLA RF.

The morphology of the three PLLA substrates observed via SEM was presented in Fig. 1. The PLLA films were quite smooth (Fig. 1A); the PLLA nanofibers were uniform and unbeaded (Fig. 1B and C). The PLLA AF were well orientated, whereas the PLLA RF had a much wider orientation distribution. The average diameter of PLLA AF was 246.71 ± 52.36 nm, and RF’s 218.57 ± 35.64 nm.

### Results of cell viability (CCK8 assay)

**Figure 2.**
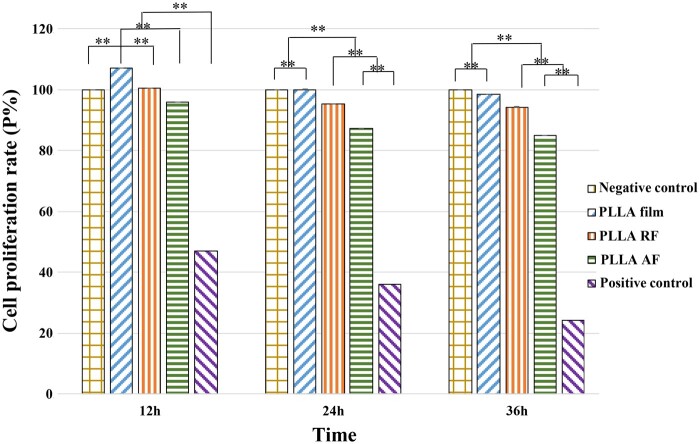
The proliferation rate of the PC12 cells on the three PLLA substrates after 12, 24 and 36 h. The negative control cells were cultured in the differentiation medium alone; the positive control cells were cultured in the medium plus 0.7% acrylamide solution without any biomaterial. All the data were presented as mean ± standard deviation. **P <* *0.01.

For the PC12 cells cultured on the three PLLA substrates after 12, 24 and 36 h, the cell viability data using CCK-8 assay were illustrated in Fig. 2. At each time point, the cell proliferation rate (P) from high to low was in order of the PLLA film groups, the RF groups, and the AF groups. The proliferation rate in each group decreased with time yet was all higher than 80% on account of the good biocompatibility of PLLA nanofibers. Mouri *et al*. [17] observed the increased differentiation rate but the decreased proliferation rate over time after culturing PC12 cells upon the exposure to nerve growth factor (NGF) for 2 days, as the number of differentiated cells exceeded that of the proliferating (undifferentiated) cells then. Our study detected the lower proliferation rate in both nanofiber groups than that of the film groups, and the lowest proliferation rate in the AF groups. This indicated that the PLLA nanofibers were more conductive to PC12 cell differentiation compared with the PLLA films, and that the AF nanofibers induced faster neuronal differentiation.

### Morphology of PC12 cells

**Figure 3.**
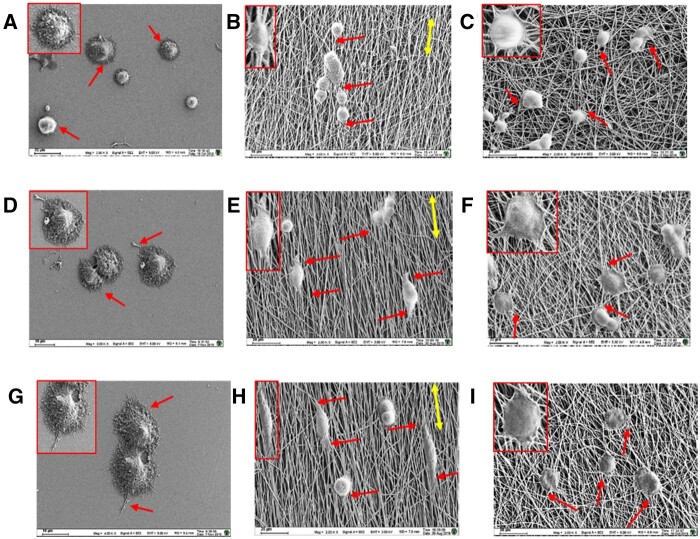
The SEM images of the PC12 cells on the three PLLA substrates after 12 h (**A–C**), 24 h (**D–F**) and 36 h (**G–I**). The red single-headed arrow represents the dendrite growth directions; the yellow double-headed arrow represents the directions of aligned nanofibers. A, D, G: the PLLA films; B, E, H: the PLLA AF; C, F, I: the PLLA RF.

The morphology of PC12 cells after being cultured on the three PLLA substrates for 12, 24 and 36 h, was displayed in Fig. 3.

The PC12 cells on the PLLA films spread neurites out randomly (Fig. 3A, D and G); however, on the PLLA AF or RF, the PC12 cells grew to follow the direction of nanofibers with extended neurites parallel to the nanofibers. The well-orientated PLLA AF induced almost the same direction of neurite growth as the direction of the aligned nanofibers with little change of neurite extension and fewer branches; the neurites extended into spindle-shaped cell bodies with time (Fig. 3B, E and H). Whereas the PLLA RF induced more neurites, since the neurites were easily affected by the contact guidance of nanofiber meshes to extend to follow the direction of adjacent nanofibers or to branch and grow out to different directions (Fig. 3C, F and I) [18].

### Characterization of neurites

For the PC12 cells cultured on the three PLLA substrates for 12, 24 and 36 h, the cell number, average number of neurites and the average neurite length per cell were analyzed by HCS/HCA and presented in Table 1.

**Table 1.** The cell number, average number of neurites and the average neurite length per cell on the three PLLA substrates (*n* = 3)

Properties	Materials	12 h	24 h	36 h
Cell number	PLLA film	483	846	1133
	PLLA AF	498	852	1116
	PLLA RF	507	827	1087
Average number of neurites	PLLA film	3.89 ± 1.84	3.17 ± 1.37	4.17 ± 1.80
	PLLA AF	2.12 ± 1.19	2.17 ± 0.97	2.58 ± 1.26
	PLLA RF	5.19 ± 2.26	4.74 ± 2.27	4.52 ± 2.00
Average length of neurites (µm)	PLLA film	5.67 ± 0.46	7.94 ± 0.72	9.78 ± 0.76
	PLLA AF	20.46 ± 0.68	23.37 ± 0.64	26.89 ± 0.52
	PLLA RF	14.75 ± 0.98	16.78 ± 0.47	19.15 ± 0.26

It could be seen from Table 1 that at the same time point, the number of cells adhered to the surface of three PLLA substrates was basically the same, but at different time points, the number of cells increased with the increase of time for all the three materials.

Neurite outgrowth underlies neuronal differentiation. During differentiation the number of neurites increases; and the neurites elongate along with certain directions according to the reception of complex intracellular and extracellular signals, thereby establishing appropriate connections with targeted neurons [19–21]. Unlike the beneficial effect of neurite elongation, neurite branching is detrimental to neural regeneration [19]. As shown in Table 1, the PC12 cells had already started differentiation after being cultured for 12 h. The average number of neurites in the AF groups was 2–3, fewer than that in the film groups (3–4) and in the RF groups (4–5), which provided least detrimental effect to neural regeneration.

Das *et al*. [22] assessed PC12 cell differentiation and neurite growth and detected that the neurites of PC12 cells after exposure to NGF for 2 days elongated with a length of at least 25 µm. As shown in Table 1, PC12 cells showed different degrees of differentiation on three different PLLA substrates. There was no induction of nanofibers on the surface of the PLLA film group, so the differentiation of PC12 cells on this surface could only be induced by NGF alone, and the inducing ability was relatively weak (the average length of neurites on PLLA film was significantly lower than that on PLLA AF and PLLA RF). While there were two inducing factors, NGF and nanofibers, in PLLA AF and PLLA RF groups, and both the differentiation degree of PC12 cells on these two nanofibers were greater than that on PLLA film induced by NGF alone. The results implied that the differentiation on PLLA AF and PLLA RF were induced by NGF and nanofibers, and the induction effect of nanofibers was greater than that of NGF (the average length of neurites in nanofiber group were more than twice that of the film group). Moreover, the PLLA AF had the highest differentiation degree (with the longest average length of neurites), indicating that the orientation of nanofibers played a leading role in the differentiation of PC12 cells.

### Analysis of gene expression

**Figure 4.**
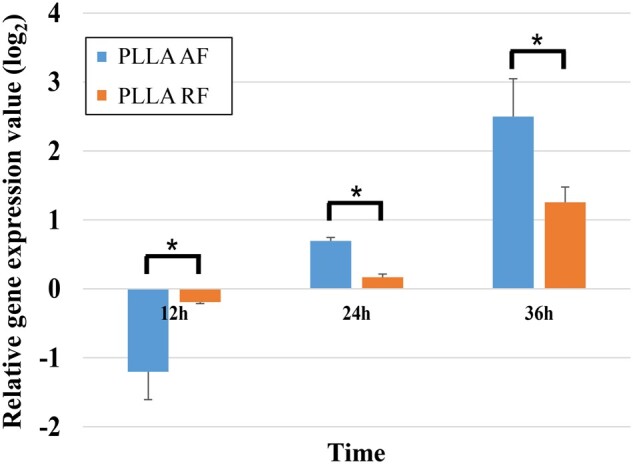
The relative *GAP43* gene expression of PC12 cells on the three PLLA substrates measured by qRT-PCR. (*P < 0.05).

The relative *GAP43* gene expression of PC12 cells cultured on the three PLLA substrates for 12 and 36 h was measured by qRT-PCR and was illustrated in Fig. 4. The *GAP*43 gene expression at the 24-h time point was discussed in our previous study [3].

The *GAP43* gene encodes the growth-associated protein 43 (GAP-43). GAP-43 is a specific presynaptic membrane protein related to the development of neurons, which only exists in neurons that have been differentiated and stereotyped and have begun to grow axons and dendrites. GAP-43 influences the axon growth ability by guiding the growth of axons and regulating the formation of new connections. The period of its high expression in the neural fiber network is closely synchronized with the extension and growth of axons and the initiation of synapse formation, so it is considered to be an internal determinant of neuron development and regeneration, and a molecular marker of neuron regeneration and plasticity [23–25].

As shown in Fig. 4, compared with the PLLA film, *GAP43* was down-regulated in the PLLA-AF-12h (AF-12h) and PLLA-RF-12h (RF-12h) groups, which was consistent with the results that *GAP*43 was down-regulated at the beginning of the differentiation of neural stems at 12 h by Lü *et al*. [23]. This might be because the differentiation had not been finalized at this time, the protrusion had just begun to grew, and the connection had not yet formed. While at 24 h [3] and 36 h, *GAP*43 in the PLLA AF and RF groups was significantly up-regulated (expression value of PLLA AF group was highest), and the expression level continued to increase over time. These results indicated that nanofibers could effectively promote the differentiation of PC12 cells during this period, manifested as axon growth and synapse formation, and the degree of promotion of PLLA AF was higher.

**Figure 5.**
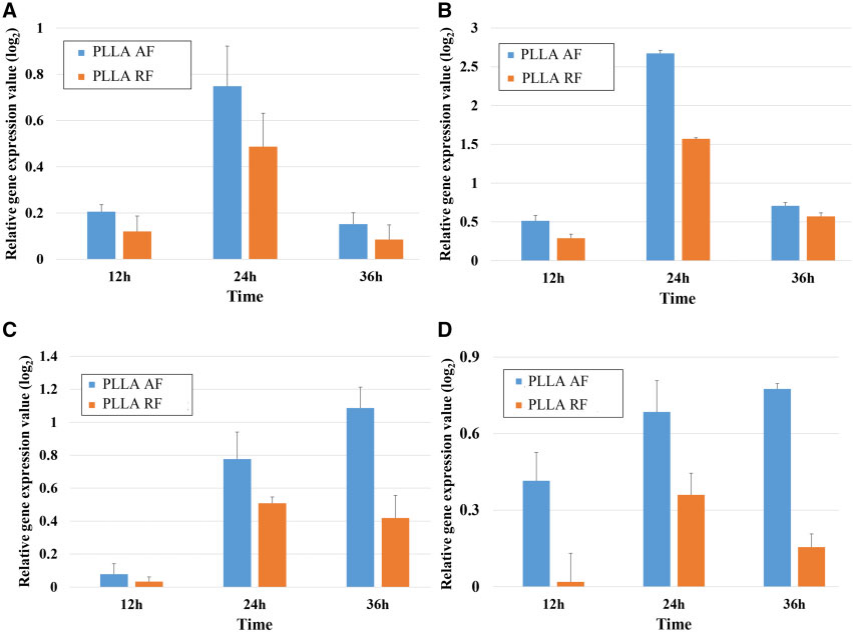
The relative gene expression of (**A**) *Pafah1b1*, (**B**) *Daam1*, (**C**) *Slit2* and (**D**) *Ascl1* in the PC12 cells on the three PLLA substrates measured by qRT-PCR.

The relative gene expression of *Pafah1b1*, *Daam1*, *Slit2* and *Ascl1* in the PC12 cells cultured on the three PLLA substrates for 12 and 36 h was measured by qRT-PCR and was illustrated in Fig. 5. The relative gene expression of these four genes at the 24-h time point was discussed in our previous study [3].

The *Pafah1b1* gene encodes the platelet-activating factor acetylhydrolase 1b regulatory subunit 1 (PAFAH1B1, also namely LIS1). *Pafah1b1* plays an important role in regulating the motor protein Dynein [26]. In addition, *Pafah1b1* genetically interacts with the *Reln* (encoding reelin) signaling pathway that modulates neuronal mitoses and migration during brain development [27]. The *Daam1* gene encodes the disheveled-associated activator of morphogenesis 1 (DAAM1). DAAM1 associates with cell motility, adhesion, cytokinesis, and other functions of the cell cortex mediated by the reorganization of actin cytoskeleton. Fen *et al*. [28] confirmed DAAM1 as an essential contributor in cell polarization regarding its effects on the actin and microtubule cytoskeleton. The *Slit2* gene encodes the slit homolog 2 protein (SLIT2). SLIT2 interacts with Glypican 1 to stabilize the cytoskeleton and control cell division [29]. The *Ascl1* gene encodes the achaete-scute homolog 1 (ASCL1), a member of the basic helix-loop-helix (BHLH) family of transcription factor. ASCL1 activates transcription by binding to an enhancer box (5'-CANNTG-3') which is involved in neuronal commitment and differentiation [30].

As shown in Fig. 5, compared with the film groups, four genes, *Pafah1b1*, *Daam1*, *Slit2* and *Ascl1*, were up-regulated in all the AF and RF groups, with higher gene expression in the former. Accordingly, the PLLA AF rather than the PLLA RF better facilitated the expression of these four genes during PC12 cell differentiation.

### Metabolomics results

#### Statistical analysis

The metabolite variability was achieved by PCA in both positive and negative ion modes (Supplementary Fig. S1). Because one repeat behaved significant divergence, the other four reproducible results in the AF-36h group were selected for analysis. For other sample groups, the data of all five repeats clustered in the score plots and thus indicated a little difference within a group. Yet the divergent clusters illustrated the metabolite differentiation among these nine sample groups.

#### Identification of differential metabolites

The types of differential metabolites in the PLLA AF and RF groups were screened by the VI*P* value of PLS-DA and the *P* values of the *t*-test (*P < 0.05*). Compared with the film groups, 51, 48 and 31 types of differential metabolites were detected at the three time points among the AF groups, respectively; and 56, 45 and 41 types among the RF groups, respectively. More detailed information of these metabolites was listed in the Supplementary Tables S1–S6. The decreased types of differential metabolites in both AF and RF groups indicated the outcome of the decreased metabolic activities in the PC12 cells as time went on. Among all the AF groups, there were similar types of differential metabolites in the AF-12h and AF-24h groups, but a significant reduction of the types in the AF-36h group. Among all the RF groups, the RF-12h group had the most types, and the RF-24h group showed a little difference from the RF-36h group.

The expression pattern was further compared as shown in Table 2. There were 38 up-regulated and 46 down-regulated differential metabolites among the AF groups, whereas 56 up-regulated and 20 down-regulated ones among the RF groups.

**Table 2.** Statistics of up-/down-regulated differential metabolites in the AF and RF groups

Change pattern		AF group	RF group
Up-regulation	Up-regulated at all 3 time points	3	21
	Up-regulated at 2 time points	14	12
	Up-regulated at 1 time point	21	23
	**Total up-regulation**	**38**	**56**
Down-regulation	Down-regulated at all 3 time points	7	4
	Down-regulated at 2 time points	12	4
	Down-regulated at 1 time point	27	12
	**Total down-regulation**	**46**	**20**

### Bioinformatics analysis of metabolomics results

#### Heat map analysis

**Figure 6.**
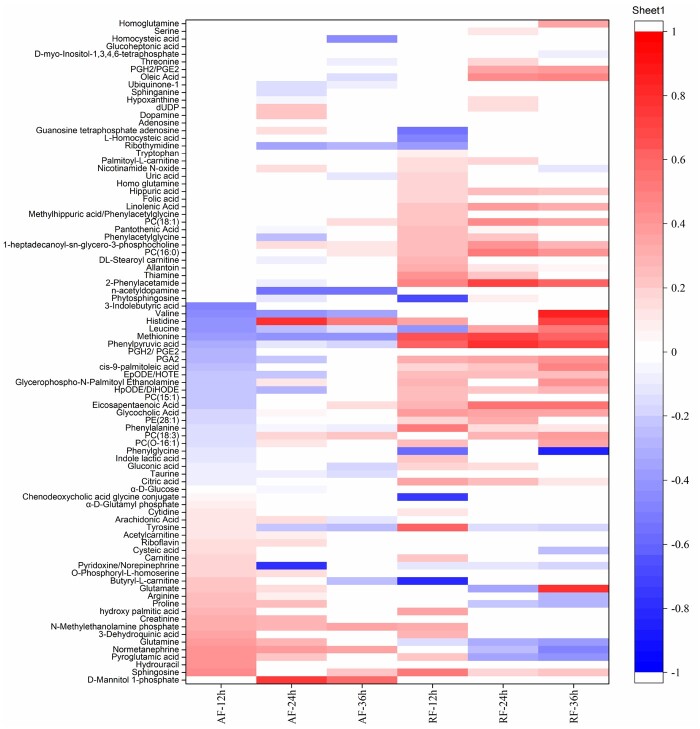
The heat map of differential metabolites among the AF and RF groups. The red color represents the metabolites with increased concentration; the blue color represents the metabolites with decreased concentration; the annotated color quantitation scale is seen as a vertical column at the right side of the heat map; the sliding color scale is representative for the degree of differentiation.

Heat map is an intuitive data visualization technique, which can group quantitative data and explore patterns to illustrate the variation. The heat map of differential metabolites among the AF and RF groups was displayed in Fig. 6, as the first step to visualize the distribution of metabolomics data. The differential metabolites among groups were highly variable. The AF groups had more types of metabolites with declined concentration compared to the RF groups. The differential metabolites between the AF-24 and AF-36h groups and between the RF-24 and RF-36h groups were the most similar.

**Figure 7.**
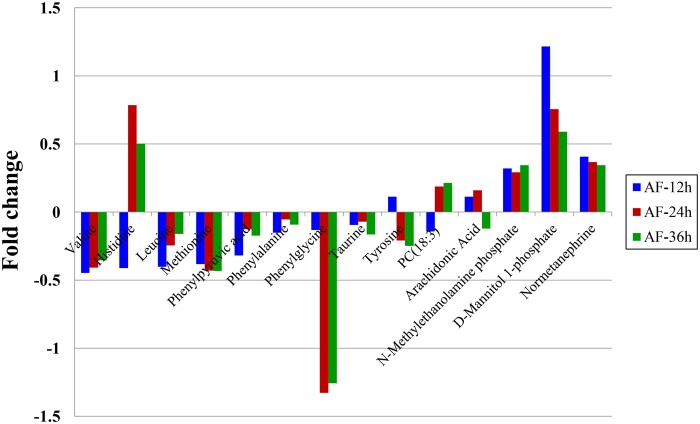
The differential metabolites at the three time points among the AF groups.

Among the AF groups, there were 14 metabolites differentially co-expressed at all three time points (Fig. 7, Supplementary Table S7) including nine amino acids, three lipids, one carbohydrate, and one neurotransmitter. The concentration of three metabolites [valine, leucine, and lysophosphatidylcholine(18:3) (LysoPC(18:3))] increased with time, while the concentration of other four metabolites (methionine, tyrosine, d-mannitol 1-phosphate and norepinephrine) decreased. The decreased concentration of nine amino acids as a whole indicated the outcome of the decreased metabolic activities associated with amino acids as time went on. As the most abundant phospholipid in the membranes of mammalian cells, LysoPC(18:3) is a phosphatidylcholine and is a precursor of the lipid second messengers like diacylglycerol and phosphatidic acid. Since the synthesis of phosphatidylcholine enhances during neurite development in neuron-like, cells such as PC12 cells [31], the increase of LysoPC(18:3) with time verified the neurite development. In addition, a sugar alcohol, d-mannitol 1-phosphate, with a big rise in concentration among all the AF groups, implied that the carbohydrate metabolism was relatively active. The increased normetanephrine suggested relatively active secretion of neurotransmitters, but its secretion was gradually reduced from 12 to 36 h.

**Figure 8.**
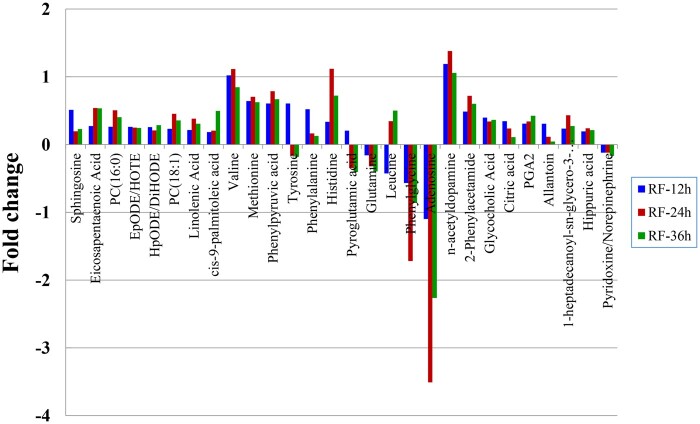
The differential metabolites at the three time points among the RF groups.

Among the RF groups, there were 28 metabolites differentially co-expressed at all three time points (Fig. 8, Supplementary Table S8) including 10 amino acids, 8 lipids, 1 nucleic acid, and 9 others. The concentration of three metabolites (*cis*-9-palmitoleic acid, leucine and prostaglandin A2) increased with time, while the concentration of other seven metabolites [epoxy-octadecadienoic acid/hydroxyoctadeca (EpODE/HOTE), tyrosine, phenylalanine, glutamine, pyroglutamic acid, citric acid and allantoin] decreased. The decrease of glutamine, an energy substrate for neurons to generate glutamate and ATP [32], indicated an increase of glutamine metabolism in the PC12 cells as time went on. Pyroglutamic acid is an endogenous cyclized derivative of glutamic acid and an intermediate of the gamma-glutamyl cycle [33], in which glutamyl moiety is cycled to pyroglutamic acid via synthesis of glutathione. Therefore, the increased concentration of pyroglutamic acid in the RF-12h group and its decrease in the RF-24h and RF-36h groups implied a decrease of glutathione metabolism over time.

### Analysis of metabolic pathways

#### Analysis of metabolic pathways relevant to differential metabolites at each time point

The differential metabolites and relevant metabolic pathways at the three time points in the AF (Supplementary Table S9) and RF (Supplementary Table S10) groups were analyzed using MetaboAnalyst. In the AF groups, the differential metabolites were involved in 35, 31 and 19 metabolic pathways at 12, 24 and 36 h, respectively. Among them, seventeen pathways were affected at all three time points (Supplementary Table S9, No. 1–17), thirteen at two time points (Supplementary Table S9, No. 18–30), and eight at only one time point (Supplementary Table S9, No. 31–38). Similarly, in the RF groups, the differential metabolites were involved in 30, 33 and 28 metabolic pathways at 12, 24 and 36 h, respectively. Among them, there were 23 (Supplementary Table S10, No. 1–23), 7 (Supplementary Table S10, No. 24–30) and 8 (Supplementary Table S10, No. 31–38) pathways affected at three, two and one time points, respectively.

#### Identification of important metabolic pathways

Among the metabolic pathways relevant to differential metabolites, the important ones at the three time points in the AF and RF groups were evaluated according to the requirement of the pathway impact greater than 0.2 or the negative log_10_ of *P* values greater than 10. So, in the AF groups (Supplementary Table S11), seven important pathways were affected at all three time points, three at two time points and two at only one time point. Similarly, in the RF groups (Supplementary Table S12), there were seven, three and three important pathways affected at three, two and one time points, respectively.

#### Identification of important metabolic pathways involved in neuronal differentiation

The metabolic pathways involved in neuronal differentiation were further identified from the important metabolic pathways (Supplementary Tables S11 and S12) based on literature, in order to investigate the influence of PLLA nanofibers on PC12 cell differentiation from the perspective of molecular level. These pathways in the AF and RF groups were listed in the Table 3.

**Table 3.** The important metabolic pathways engaged in neuronal differentiation and the involvement of the differential metabolites in the PLLA AF and RF groups

No.	Important metabolic pathways for neuronal differentiation	PLLA AF			PLLA RF		
		12h	24h	36h	12h	24h	36h
1	Phenylalanine, tyrosine and tryptophan biosynthesis	√	√	√	√	√	√
2	Phenylalanine metabolism	√	√	√	√	√	√
3	Histidine metabolism	√	√	√	√	√	√
4	Aminoacyl-tRNA biosynthesis	√	√	√	√	√	√
5	Alanine, aspartate and glutamate metabolism	√	√	–	–	√	√
6	d-glutamine and d-glutamate metabolism	√	√	–	–	√	√
7	Taurine and hypotaurine metabolism	√	√	√	–	–	–
8	Arachidonic acid metabolism	√	√	√	–	–	–
9	Tyrosine metabolism		√	–	–	–	–
10	Glycine, serine and threonine metabolism	–	–	–	–	√	–

√ represents the involvement of the differential metabolites; - represents the noninvolvement.

Ten significant metabolic pathways in total, including nine in the AF groups and seven in the RF groups, were engaged in neuronal differential as shown in Table 3. Among them, six pathways were mutual in both AF and RF groups (Table 3, No. 1–6); three pathways were specific to the AF groups (Table 3, No. 7–9) and one specific to the RF groups (Table 3, No. 10). These 10 metabolic pathways could play a critical role in PC12 cell differentiation induced by the PLLA nanofibers.

#### Identification of metabolic pathways and metabolites critical to PC12 cell differentiation

**Figure 9.**
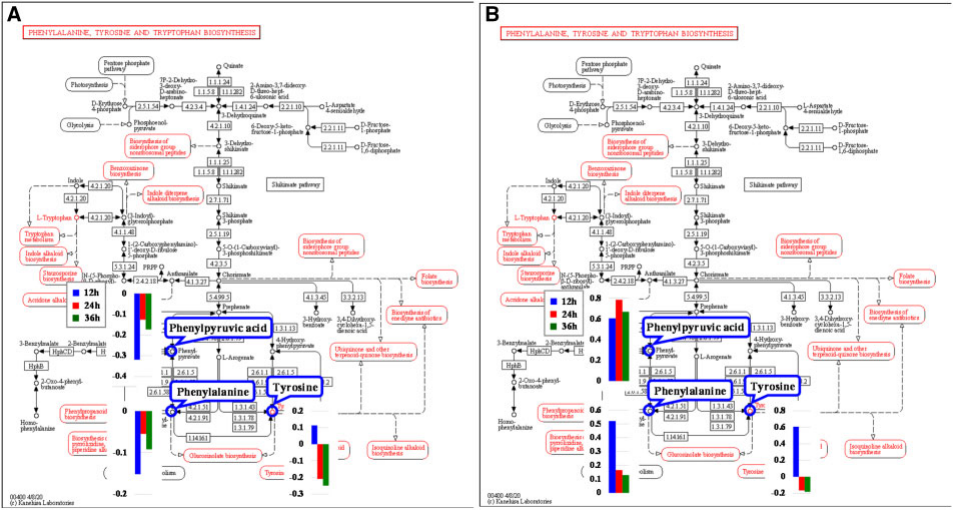
The phenylalanine, tyrosine and tryptophan biosynthesis pathway [34] and relevant metabolites in the (a) PLLA AF and (b) PLLA RF groups.

**Figure 10.**
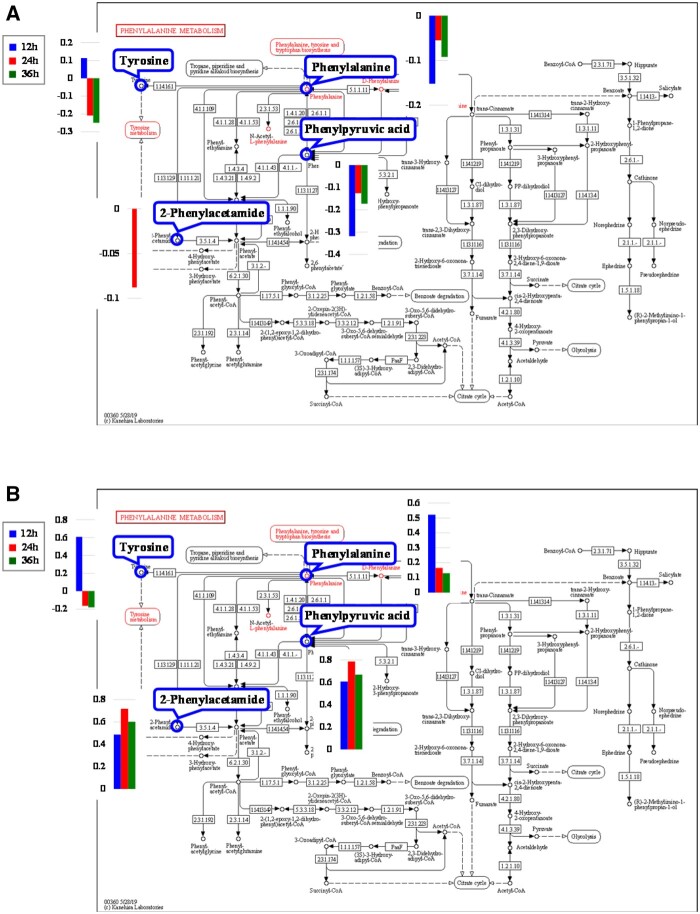
The phenylalanine metabolism pathway [34] and relevant metabolites in the (**A**) PLLA AF and (**B**) PLLA RF groups.

According to the first two criteria discussed in Section ‘Identification of metabolic pathways and metabolites critical to PC12 cell differentiation influenced by PLLA nanofibers’, six pathways in the AF groups (Table 3, No. 1–6) and four in the RF groups (Table 3, No. 1–4) were determined. In addition, only the phenylalanine, tyrosine and tryptophan biosynthesis (Fig. 9) and the phenylalanine metabolism (Fig. 10) met the third criterion. The relevant differential metabolites and their concentration level were listed in Tables 4 and 5.

**Table 4:** The critical metabolic pathways and relevant differential metabolites

No.	Pathway	PLLA AF			PLLA RF		
		12 h	24 h	36 h	12 h	24 h	36 h
1	Phenylalanine, tyrosine and tryptophan biosynthesis	Phenylalanine Phenylpyruvic acid Tyrosine	Phenylalanine Phenylpyruvic acid Tyrosine	Phenylalanine Phenylpyruvic acid Tyrosine	Phenylalanine Phenylpyruvic acid Tyrosine	Phenylalanine Phenylpyruvic acid Tyrosine	Phenylalanine Phenylpyruvic acid Tyrosine
2	Phenylalanine metabolism	Phenylalanine Phenylpyruvic acid Tyrosine	Phenylalanine Phenylpyruvic acid Tyrosine 2-Phenylacetamide	Phenylalanine Phenylpyruvic acid Tyrosine	Phenylalanine Phenylpyruvic acid Tyrosine 2-Phenylacetamide	Phenylalanine Phenylpyruvic acid 2-Phenylacetamide	Phenylalanine Phenylpyruvic acid 2-Phenylacetamide

Both these two metabolic pathways met the fourth criterion, because the two metabolites, phenylalanine and phenylpyruvic acid, were involved in each pathway and showed the same pattern of differential change at all three time points in the AF groups. Whereas, in the RF groups, 2-phenylacetamide was an additional metabolite besides phenylalanine and phenylpyruvic acid. Therefore, the phenylalanine, tyrosine and tryptophan biosynthesis pathway (Fig. 9) and the phenylalanine metabolism pathway (Fig. 10) were identified as critical metabolic pathways.

The critical metabolites were selected according to the criteria in Section ‘Identification of metabolic pathways and metabolites critical to PC12 cell differentiation influenced by PLLA nanofibers’: phenylalanine, phenylpyruvic acid and tyrosine in the AF groups; and phenylalanine, phenylpyruvic acid, tyrosine and 2-phenylacetamide in the RF groups.

##### Phenylalanine, tyrosine and tryptophan biosynthesis

Three critical metabolites, phenylalanine, phenylpyruvic acid and tyrosine, were engaged in the phenylalanine, tyrosine and tryptophan biosynthesis in both AF and RF groups (Fig. 9).

As an essential amino acid in humans, phenylalanine plays a key role in the biosynthesis of other amino acids and is important in the structure and function of many proteins and enzymes [35]. Phenylalanine can be converted into tyrosine [36]. The defective conversion results in toxic concentrations of phenylalanine in the brain tissue and cerebrospinal fluid, which is the cause of phenylketonuria. The urine of phenylketonuria patients thereby contains large amounts of phenylalanine, phenylpyruvic acid, phenylacetic acid and phenyllactic acid [37], as phenylalanine can be converted into phenylpyruvic acid and vice versa under certain condition. Phenylketonuria is neuropathologically characterized by reduced dendritic arborization, loss of synapses and neurodegeneration. Previous studies have revealed that high concentration of phenylalanine and its metabolites have adverse effect on nerve cell growth and cell differentiation [38], for example, damage to the central nervous system [39, 40], increased death of hippocampal neurons and primary cultured neurons isolated from mouse embryonic cortex [41] and impaired synaptogenesis in phenylketonuria with a decreased synaptic density in phenylalanine-treated cortical culture [38].

Tyrosine is a nonessential amino acid that the body makes from phenylalanine. As a precursor of neurotransmitters like norepinephrine and dopamine, tyrosine can be used as a mood enhancer and antidepressant; it also improves memory and alertness [42].

In this article, the concentration of phenylalanine and phenylpyruvic acid decreased in the AF groups but increased in the RF groups. The concentration of tyrosine in both AF and RF groups increased at 12 h and decreased at 24 h with further decrease at 36 h. In the AF groups, the phenylalanine could be used for the biosynthesis of dopamine and norepinephrine due to the decreased phenylacetic acid and the less phenylalanine converting into tyrosine; and this thereby contributed to promoting PC12 cell differentiation on the aligned nanofibers. Whereas in the RF groups, the accumulation of phenylalanine and phenylpyruvic acid might have negative effect on PC12 cell differentiation.

##### Phenylalanine metabolism

Four critical metabolites, phenylalanine, phenylpyruvic acid, tyrosine and 2-phenylacetamide, were engaged in the phenylalanine, tyrosine and tryptophan biosynthesis in both AF and RF groups (Fig. 10).

Phenylalanine can be converted into 2-phenylacetamide, which is the direct precursor of the neuromodulator phenethylamine. Phenethylamine is an excitatory monoamine neurotransmitter produced from phenylalanine via enzymatic decarboxylation in the central nervous system. Phenethylamine not only increases extracellular dopamine [43] but also leads inhibitory responses in dopaminergic neurons [44]. In our study, the concentration of 2-phenylacetamide decreased in the AF-24h group but increased in all RF groups. This suggested that with lower conversion rate to 2-phenylacetamide, phenylalanine mainly converted into tyrosine to promote the biosynthesis of dopamine and norepinephrine in the AF groups. Whereas the more active 2-phenylacetamide metabolism could facilitate the biosynthesis of phenethylamine with declined dopamine, thus impairing PC12 cell differentiation.

#### Identification of genes/proteins that regulate critical metabolites

The genes and proteins involved in the two abovementioned critical metabolic pathways were compared with the differentially expressed genes at 24-h time point [3] and proteins in our previous studies, respectively. Three differentially expressed genes in the AF groups and one in the RF groups were involved in the two critical pathways. Only one gene, *Lao1*, was relevant to the critical metabolites (Table 6). No differentially expressed proteins participated in these two pathways.

**Table 6.** The gene involved in the two critical metabolic pathways in the PC12 cells cultured on the PLLA nanofibers for 24 h

No.	Pathway	PLLA AF		PLLA RF	
		Gene	Expression level (log_2_)	Gene	Expression level (log_2_)
1	Phenylalanine, tyrosine, and tryptophan biosynthesis	Lao1↓	−1.22	Lao1↓	−0.81
2	phenylalanine metabolism				

*Lao1* (Figs 9 and 10), corresponding to the enzyme 1.4.3.2 (l-amino acid oxidase), is directly related to phenylalanine and phenylpyruvic acid. *Lao1* encodes an amino acid oxidase, LAO1, which can oxidize phenylpyruvic acid to phenylalanine. In the AF groups, the down-regulation (−1.12) of *Lao1* prevented phenylpyruvic acid (−0.126) from converting into phenylalanine, which caused the decrease of phenylalanine (−0.055) and its downstream metabolites like tyrosine (−0.209) and 2-phenylacetamide (−0.088) (Table 7). Despite the increased concentration in the RF groups, the much less phenylalanine than phenylpyruvic acid implied the inhibitory effect of the down-regulated *Lao1* on phenylpyruvic acid converting into phenylalanine, which thereby led to the declined concentration of its downstream metabolite, tyrosine (−0.165).

**Table 7.** The comparison of the PLLA AF and RF influencing PC12 cell differentiation

	PLLA AF	PLLA RF
Metabolic pathways important to neuronal differentiation	6 mutual, 3 specific	6 mutual, 1 specific
Relevant differential metabolites of important metabolic pathways	14 mutual, 5 specific	14 mutual, 1 specific
Critical metabolic pathways	2 mutual	
	① Phenylalanine, tyrosine, and tryptophan biosynthesis pathway	
	② Phenylalanine metabolism pathway	
Critical metabolites	①phenylalanine ↓	①phenylalanine ↑
	②phenylpyruvic acid ↓	②phenylpyruvic acid ↑
	③tyrosine ↓	③tyrosine ↓
		④22-phenylacetamide ↑
Differential expression of the neuronal differentiation marker gene and the genes relevant to neurite outgrowth	High	Low
Differential expression of the genes that regulated critical metabolites in cell differentiation	High	Low
Cell proliferation rate	Low	High
Neurite length (beneficial for cell differentiation)	Long	Short
Number of neurites (less effective for cell differentiation)	Small number	Large number
Impact on cells	More beneficial to cell differentiation	Less effective for cell differentiation

↑ represents increased concentration. ↓ represents decreased concentration.

### Verification of the differentiation of metabolites and the functions of critical metabolic pathways

#### Verification of the differentiation of critical metabolites

The concentration of two critical metabolites, phenylalanine and tyrosine, was performed using targeted metabolomics LC-MS (Supplementary Fig. S2). This was similar to the result by non-targeted metabolomics LC-MS. The phenylalanine decreased in the AF groups (relative change >0) while increased in the RF groups (relative change <0). The tyrosine increased at 12 h but decreased at 24 h and 36 h in both AF and RF groups.

#### Verification of the functions of critical metabolic pathways

The phenylalanine was involved in the two critical metabolic pathways in the AF and RF groups. Zhang *et al*. [45] found that the high concentration of phenylalanine was capable of increasing the intracellular Ca^2+^ concentration via two mechanisms. It enhanced the gene expression of *Cacnald* (GenBank^TM^ D38101) and *Cacnb3* (GenBank^TM^ M88751) of the voltage-dependent L-type calcium channel, thus leading to an influx of Ca^2+^ into the cell. In the meantime, it reduced the expression of plasma membrane Ca^2+^ ATPase (PMCA) 2a and PMCA 2b which served to remove Ca^2+^ from the cell.

**Figure 11.**
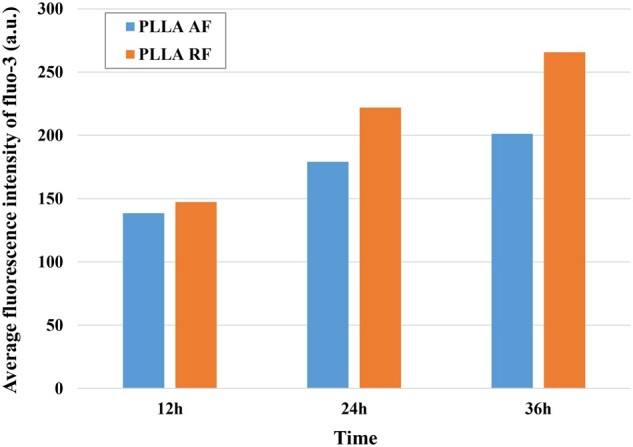
The average fluorescence intensity of intracellular fluo-3 on the three substrates at each time point.

Fluo-3 AM, the acetoxymethyl (AM) derivative of fluo-3, was a fluorescent Ca^2+^ indicator and able to assist fluo-3 to penetrate cell membrane. Once inside the cell, unspecific esterases cleave the ester effectively trapping fluo-3 [46]. Despite a non-fluorescent compound itself, fluo-3 exhibits large green-fluorescence intensity on binding Ca^2+^, which allows the detection of intracellular Ca^2+^ concentration. The average fluorescence intensity of intracellular fluo-3 on the three substrates were illustrated in Fig. 11. At each time point, the fluorescence intensity in the RF groups was higher than that in the AF groups, respectively. This was consistent with the higher phenylalanine concentration in the AF groups discussed in the metabolomics experiment.

Calcium ion influx initiates a cascade of events including activation of the Ras-MAPK signaling cascade, stimulation of calcium/calmodulin and calmodulin kinase signaling pathway, activation of Rac GTPase, and transcription of hundreds of genes. These regulatory activities promote many diverse processes, such as neuronal survival, dendrite formation, synaptic development and adaptive responses [47]. Aizawa *et al*. [48] investigated a calcium-responsive transactivator (CREST) and its role in supporting neuronal morphogenesis. Barbosa *et al*. [49] observed the distinct function of a calcium-dependent regulator, myocyte enhancer factor 2 (MEF2), to regulate synapse and the efficiency of synaptic transmission.

The PLLA AF contributed to an increase of phenylalanine concentration in the PC12 cells (Table 5). The rise of phenylalanine enabled more Ca^2+^ influx by regulating the calcium channel and reducing Ca^2+^-ATPase (Fig. 11). As a result, the increased intracellular Ca^2+^ concentration led to the up-regulated expression of important regulators on dendrite arborization and synapse number like CREST and MEF2 [3], thus leading to the increased neurite branching. This was consistent with the more neurites in the PC12 cells on the AF surface than those on the RF surface (Table 1).

**Table 5.** The relevant differential metabolites and their concentration level (log_2_) involved in critical metabolic pathways

No.	Differential metabolites	PLLA AF			PLLA RF		
		12 h	24 h	36 h	12 h	24 h	36 h
1	Phenylalanine	−0.152	−0.055	−0.092	0.522	0.165	0.129
2	Phenylpyruvic acid	−0.319	−0.126	−0.173	0.607	0.786	0.670
3	Tyrosine	0.113	−0.209	−0.249	0.607	−0.165	−0.180
4	2-Phenylacetamide		−0.088		0.487	0.719	0.602

**Figure 12.**
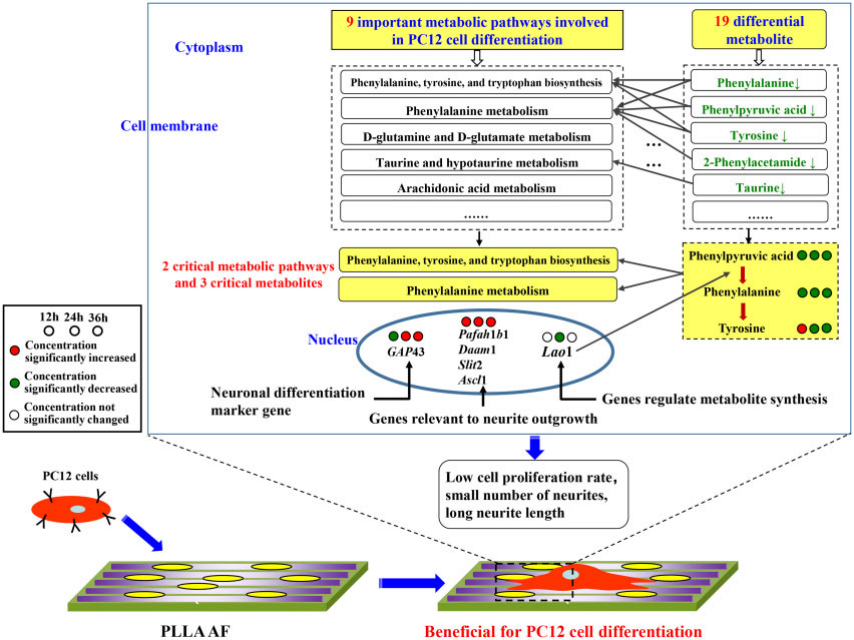
The molecular mechanism of PLLA AF on PC12 cell differential at metabolic level.

In summary, in the PC12 cells growing on the surface of the PLLA nanofibers, the metabolites of differentiation-related metabolic pathways underwent a series of enzymatic reactions; thereby the quantitation of these metabolites as reactants, products, and intermediates changed with time. The molecular mechanism of PLLA AF on PC12 cell differential at metabolic level was diagramed in Fig. 12. The factors that affect the differentiation of neurons include biochemical inducers, physical stimuli and biomaterials. NGF belongs to biochemical inducers while PLLA AF is one kind of biomaterial. Therefore, their inducing roles for PC12 cells differentiation were not exactly the same in several aspects, including the recognition substances on the cell membrane surface, the triggered intracellular signal molecules, the mainly affected signal pathways and the overall induction mechanism [3, 50–54]. With cDNA microarray, bioinformatics analysis and verification experiments, our research group [3] further found that PLLA AF might be sensed by PC12 cells *via* the combination of integrins and adsorbed proteins. This combination triggers the activation of integrin-mediated FAK-MEK-ERK pathway, regulation of actin cytoskeleton pathway, focal adhesion pathway and MAPK signaling pathway. The *Pafah*1*b*1 gene cooperating with NGF to facilitate PC12 cell differentiation.

The impacts of PLLA AF and RF on PC12 cell differentiation were compared in Table 7.

In the AF groups, nine important metabolic pathways were involved in neuronal differentiation with the concentration change of nineteen metabolites over the three time points, of which three and five were AF-specific, respectively. Among them, the two critical pathways were the phenylalanine, tyrosine, and tryptophan biosynthesis and the phenylalanine metabolism with three reduced critical metabolites, phenylalanine, phenylpyruvic acid, and tyrosine. In the process of PC12 cell differentiation facilitated by the PLLA AF, the differential metabolites support cell differentiation through their corresponding metabolic pathways, including the up-regulation of the neuronal differentiation marker gene and the genes relevant to neurite outgrowth and the down-regulation of *Lao1* for the synthesis of phenylalanine, resulting in lower cell proliferation rate and a smaller number of longer neurites. Therefore, the PLLA AF were beneficial for cell differentiation.

In the RF groups, seven important metabolic pathways were involved in neuronal differentiation with the concentration change of fifteen metabolites over the three time points, of which one and one were RF-specific, respectively. Among them, the same two critical pathways as the AF groups included four critical metabolites, phenylalanine, phenylpyruvic acid, 2-phenylacetamide, and tyrosine. The first three metabolites increased, and tyrosine decreased as time went on. The inhibition of phenylalanine–tyrosine conversion yielded mainly phenylpyruvic acid and 2-phenylacetamide as well as the downstream phenethylamine, whose over-accumulation was detrimental to PC12 cell differentiation [44, 55]. Compared with the AF groups, the PLLA RF were less effective regulating the gene expression, concentration of the metabolites, and relevant metabolic pathways essential to cell differentiation; the lower gene expression of the relevant genes and *Lao1* in the latter led to higher cell proliferation rate and a larger number of shorter neurites.

## Conclusion

In this article, the metabolomics approach was used for the first time to study the influence of the orientation of nanofibers on neuronal differentiation. The two metabolic pathways and three metabolites were identified as critical to neuronal differentiation induced by the AF and RF. These metabolomics results, combining with the differential expression analysis of the neuronal differentiation marker gene and the genes relevant to neurite outgrowth, then demonstrated the different molecular mechanisms influenced by the AF and RF. As a result, the PLLA AF were more beneficial for PC12 cell differentiation with a smaller number of longer neurites; whereas the PLLA RF were less effective with a larger number of shorter neurites.

## Supplementary data

Supplementary data are available at *REGBIO* online.

## Supplementary Material

rbab031_Supplementary_Data
