# Supplementary material for: Metabolomics analysis of poly(l-lactic acid) nanofibers' performance on PC12 cell differentiation
Source: Regen Biomater. 2021 Jun 21;8(4):rbab031. doi: 10.1093/rb/rbab031 (PMC8218933; doi:10.1093/rb/rbab031)
Supplement: rbab031_Supplementary_Data [file rbab031_supplementary_data.docx]

**Supporting Information**

**Title:** Metabolomics analysis of poly(L-lactic acid) nanofibers' performance on PC12 cell differentiation

**Author:** Xiaoman Su^1,#^, Yan Huang^1,#^, Rong Chen^1^, Yiwen Zhang^2^, Meichen He^1^, Xiaoying Lü^1,*^

**Supplementary Table S1:** Differentially metabolites in PC12 cells cultured on PLLA AF after 12 h

**Supplementary Table S2:** Differentially metabolites in PC12 cells cultured on PLLA AF after 24 h

**Supplementary Table S3:** Differentially metabolites in PC12 cells cultured on PLLA AF after 36 h

**Supplementary Table S4:** Differentially metabolites in PC12 cells cultured on PLLA RF after 12 h

**Supplementary Table S5:** Differentially metabolites in PC12 cells cultured on PLLA RF after 24 h

**Supplementary Table S6:** Differentially metabolites in PC12 cells cultured on PLLA RF after 36 h

**Supplementary Table S7:** Differentially mutual metabolites in PC12 cells cultured on PLLA AF for 12, 24 and 36 h

**Supplementary Table S8:** Differentially mutual metabolites in PC12 cells cultured on PLLA RF for 12, 24 and 36h

**Supplementary Table S9:** Metabolic pathways that differentially metabolites in PC12 cells cultured on PLLA AF after 12, 24 and 36 h involved in

**Supplementary Table S10:** Metabolic pathways that differentially metabolites in PC12 cells cultured on PLLA RF after 12, 24 and 36 h involved in

**Supplementary Table S11:** Important metabolic pathways and pathway impact in PLLA AF groups

**Supplementary Table S12:** Important metabolic pathways and pathway impact in PLLA RF groups

**Supplementary Figure S1:** The metabolite variability achieved by PCA in both positive (a) and negative (b) ion modes.

**Supplementary Figure S2:** The relative concentration change of phenylalanine (a) and tyrosine (b).

**Supplementary Table S1:** Differentially metabolites in PC12 cells cultured on PLLA AF after 12 h.

| NO. | VIP | mz | rt | Metabolite | p value | Relative change (log_2_) |
| --- | --- | --- | --- | --- | --- | --- |
| 1 | 1.432 | 318.2998 | 8.32 | Phytosphingosine | 0 | 1.052 |
| 2 | 1.359 | 204.0999 | 1.15 | 3-Indolebutyric acid | 0 | 0.492 |
| 3 | 1.125 | 118.0862 | 1.1 | Valine | 0.007 | 0.444 |
| 4 | 1.35 | 132.1013 | 1.31 | Leucine | 0 | 0.403 |
| 5 | 1.153 | 156.0764 | 1.15 | Histidine | 0.006 | 0.397 |
| 6 | 1.165 | 150.058 | 1.12 | Methionine | 0.004 | 0.38 |
| 7 | 1.046 | 165.0545 | 1.17 | Phenylpyruvic acid | 0.016 | 0.319 |
| 8 | 1.277 | 335.2185 | 10.61 | PGA2 | 0.001 | 0.297 |
| 9 | 2.14 | 271.2288 | 12.974 | hydroxy palmitic acid | 0 | 0.29 |
| 10 | 1.007 | 353.229 | 9.3 | PGH2/PGE2 | 0.035 | 0.281 |
| 11 | 1.083 | 255.2312 | 11.36 | cis-9-palmitoleic acid | 0.012 | 0.249 |
| 12 | 1.195 | 295.2261 | 10.61 | EpODE/HOTE | 0.003 | 0.228 |
| 13 | 1.21 | 313.2367 | 10.61 | HpODE/DiHODE | 0.002 | 0.222 |
| 14 | 1.095 | 454.2915 | 11.01 | Glycerophospho-N-Palmitoyl Ethanolamine | 0.011 | 0.221 |
| 15 | 1.226 | 482.323 | 12.19 | PC(15:1) | 0.002 | 0.22 |
| 16 | 1.02 | 303.2313 | 11.59 | Eicosapentaenoic Acid | 0.03 | 0.202 |
| 17 | 1.102 | 634.4514 | 12.28 | PE(28:1) | 0.009 | 0.173 |
| 18 | 1.001 | 518.3203 | 11.09 | PC(18:3) | 0.039 | 0.139 |
| 19 | 1.007 | 480.3427 | 11.43 | PC(O-16:1) | 0.023 | 0.133 |
| 20 | 1.279 | 152.0736 | 1.32 | Phenylglycine | 0.001 | 0.131 |
| 21 | 1.066 | 466.3154 | 8.18 | Glycocholic Acid | 0.014 | 0.124 |
| 22 | 1.842 | 303.2341 | 13.428 | Arachidonic Acid | 0.01 | 0.115 |
| 23 | 1.641 | 226.0129 | 1.131 | α-D-Glutamyl phosphate | 0.032 | 0.091 |
| 24 | 1.812 | 448.3083 | 9.177 | Chenodeoxycholic acid glycine conjugate | 0.012 | 0.06 |
| 25 | 1.151 | 182.0809 | 0.83 | Tyrosine | 0.006 | -0.0075 |
| 26 | 1.756 | 179.057 | 0.764 | α-D-Glucose | 0.017 | -0.024 |
| 27 | 1.85 | 191.0203 | 1.166 | Citric acid | 0.009 | -0.08 |
| 28 | 1.635 | 124.0081 | 0.762 | Taurine | 0.033 | -0.094 |
| 29 | 2.023 | 195.0522 | 0.782 | Gluconic acid | 0.002 | -0.098 |
| 30 | 1.254 | 244.0998 | 2.14 | Cytidine | 0.001 | -0.101 |
| 31 | 1.126 | 204.1276 | 0.82 | Acetylcarnitine | 0.007 | -0.118 |
| 32 | 1.662 | 204.0671 | 5.938 | Indolelactic acid | 0.029 | -0.13 |
| 33 | 1.109 | 377.1446 | 4.99 | Riboflavin | 0.01 | -0.148 |
| 34 | 1.995 | 164.072 | 2.097 | Phenylalanine | 0.003 | -0.15 |
| 35 | 1.106 | 170.011 | 0.6 | Cysteic acid | 0.009 | -0.16 |
| 36 | 1.275 | 162.1122 | 0.78 | Carnitine | 0.001 | -0.171 |
| 37 | 1.317 | 170.0812 | 0.83 | Pyridoxine/Norepinephrine | 0 | -0.19 |
| 38 | 1.141 | 200.0359 | 0.71 | O-Phosphoryl-L-homoserine | 0.008 | -0.198 |
| 39 | 1.286 | 232.1537 | 2.76 | Butyryl-L-carnitine | 0.001 | -0.212 |
| 40 | 1.071 | 148.0633 | 0.8 | Glutamate | 0.013 | -0.219 |
| 41 | 1.31 | 116.0706 | 0.79 | Proline | 0 | -0.261 |
| 42 | 1.301 | 175.1179 | 0.66 | Arginine | 0 | -0.262 |
| 43 | 1.357 | 156.0432 | 0.77 | N-Methylethanolamine phosphate | 0 | -0.321 |
| 44 | 1.175 | 114.0662 | 0.77 | Creatinine | 0.004 | -0.325 |
| 45 | 1.394 | 191.0508 | 0.74 | 3-Dehydroquinic acid | 0 | -0.365 |
| 46 | 1.403 | 147.0763 | 0.8 | Glutamine | 0 | -0.371 |
| 47 | 1.328 | 184.094 | 0.75 | Normetanephrine | 0 | -0.409 |
| 48 | 1.392 | 130.0497 | 0.8 | Pyroglutamic acid | 0 | -0.413 |
| 49 | 1.332 | 129.0656 | 0.78 | Hydrouracil | 0 | -0.419 |
| 50 | 1.274 | 300.289 | 9.41 | Sphingosine | 0.001 | -0.45 |
| 51 | 1.356 | 263.0468 | 0.79 | D-Mannitol 1-phosphate | 0 | -1.222 |

**Supplementary Table S2:** Differentially metabolites in PC12 cells cultured on PLLA AF after 24 h.

| NO. | VIP | mz | Rt | Metabolite | p value | Relative change (log_2_) |
| --- | --- | --- | --- | --- | --- | --- |
| 1 | 1.297 | 156.0764 | 1.15 | Histidine | 0.001 | 0.808 |
| 2 | 1.244 | 263.0468 | 0.79 | D-Mannitol 1-phosphate | 0.003 | 0.758 |
| 3 | 1.403 | 184.094 | 0.75 | Normetanephrine | 0 | 0.369 |
| 4 | 1.397 | 147.0763 | 0.8 | Glutamine | 0 | 0.3 |
| 5 | 1.449 | 156.0432 | 0.77 | N-Methylethanolamine phosphate | 0 | 0.293 |
| 6 | 1.218 | 114.0662 | 0.77 | Creatinine | 0.004 | 0.286 |
| 7 | 1.387 | 116.0706 | 0.79 | Proline | 0 | 0.238 |
| 8 | 1.164 | 154.0835 | 1.31 | Dopamine | 0.009 | 0.234 |
| 9 | 1.105 | 389.0091 | 0.67 | dUDP | 0.018 | 0.212 |
| 10 | 1.31 | 518.3203 | 11.09 | PC(18:3) | 0.001 | 0.187 |
| 11 | 1.322 | 148.0633 | 0.8 | Glutamate | 0.001 | 0.166 |
| 12 | 1.39 | 305.2484 | 13.45 | Arachidonic Acid | 0 | 0.16 |
| 13 | 1.2 | 139.0531 | 0.8 | Nicotinamide N-oxide | 0.005 | 0.158 |
| 14 | 1.322 | 377.1446 | 4.99 | Riboflavin | 0.001 | 0.155 |
| 15 | 1.142 | 200.0359 | 0.71 | O-Phosphoryl-L-homoserine | 0.011 | 0.151 |
| 16 | 1.282 | 510.3543 | 11.68 | 1-heptadecanoyl-sn-glycero-3-phosphocholine | 0.002 | 0.15 |
| 17 | 1.097 | 853.0299 | 6.25 | Guanosine tetraphosphate adenosine | 0.017 | 0.148 |
| 18 | 1.127 | 454.2915 | 11.01 | Glycerophospho-N-Palmitoyl Ethanolamine | 0.013 | 0.134 |
| 19 | 1.159 | 480.3427 | 11.43 | PC(O-16:1) | 0.009 | 0.131 |
| 20 | 1.047 | 175.1179 | 0.66 | Arginine | 0.032 | 0.099 |
| 21 | 1.151 | 204.1276 | 0.82 | Acetylcarnitine | 0.009 | 0.098 |
| 22 | 1.228 | 130.0497 | 0.8 | Pyroglutamic acid | 0.004 | 0.081 |
| 23 | 1.798 | 464.3031 | 8.18 | Glycocholic Acid | 0.015 | 0.055 |
| 24 | 1.001 | 205.0961 | 3.88 | Tryptophan | 0.045 | -0.031 |
| 25 | 1.601 | 179.057 | 0.76 | α-D-Glucose | 0.04 | -0.036 |
| 26 | 1.156 | 220.1176 | 2.76 | Pantothenic Acid | 0.01 | -0.042 |
| 27 | 1.696 | 135.0306 | 0.8 | Hypoxanthine | 0.026 | -0.048 |
| 28 | 1.895 | 124.0081 | 0.76 | Taurine | 0.008 | -0.071 |
| 29 | 1.132 | 166.0852 | 2.14 | Phenylalanine | 0.013 | -0.079 |
| 30 | 1.156 | 136.0756 | 1.17 | 2-Phenylacetamide | 0.01 | -0.087 |
| 31 | 1.035 | 428.372 | 11.04 | DL-Stearoylcarnitine | 0.029 | -0.098 |
| 32 | 1.243 | 165.0545 | 1.17 | Phenylpyruvic acid | 0.003 | -0.125 |
| 33 | 1.373 | 318.2998 | 8.32 | Phytosphingosine | 0 | -0.133 |
| 34 | 1.126 | 302.3048 | 9.11 | Sphinganine | 0.012 | -0.137 |
| 35 | 2.14 | 249.1142 | 8.88 | Ubiquinone-1 | 0.001 | -0.138 |
| 36 | 1.381 | 295.2261 | 10.61 | EpODE/HOTE | 0 | -0.201 |
| 37 | 1.386 | 182.0804 | 1.17 | Tyrosine | 0 | -0.208 |
| 38 | 1.382 | 335.2185 | 10.61 | PGA2 | 0 | -0.218 |
| 39 | 1.369 | 132.1013 | 1.31 | Leucine | 0 | -0.245 |
| 40 | 1.142 | 194.0841 | 1.15 | Phenylacetylglycine | 0.01 | -0.249 |
| 41 | 1.37 | 313.2367 | 10.61 | HpODE/DiHODE | 0 | -0.266 |
| 42 | 1.374 | 259.0924 | 1.14 | Ribothymidine | 0 | -0.356 |
| 43 | 1.417 | 118.0862 | 1.1 | Valine | 0 | -0.406 |
| 44 | 1.443 | 150.058 | 1.12 | Methionine | 0 | -0.427 |
| 45 | 1.399 | 196.1 | 1.1 | n-acetyldopamine | 0 | -0.529 |
| 46 | 1.469 | 170.0813 | 1.12 | Pyridoxine/Norepinephrine | 0 | -0.77 |
| 47 | 1.488 | 152.0736 | 1.32 | Phenylglycine | 0 | -1.327 |
| 48 | 1.449 | 268.1038 | 1.16 | Adenosine | 0 | -2.706 |

**Supplementary Table S3:** Differentially metabolites in PC12 cells cultured on PLLA AF after 36 h.

| NO. | VIP | mz | Rt | Metabolite | p value | Relative change (log_2_) |
| --- | --- | --- | --- | --- | --- | --- |
| 1 | 1.346 | 263.0468 | 0.79 | D-Mannitol 1-phosphate | 0.018 | 0.591 |
| 2 | 1.208 | 156.0764 | 1.15 | Histidine | 0.046 | 0.568 |
| 3 | 1.474 | 184.094 | 0.75 | Normetanephrine | 0.006 | 0.351 |
| 4 | 1.675 | 156.0432 | 0.77 | N-Methylethanolamine phosphate | 0 | 0.347 |
| 5 | 1.418 | 300.289 | 9.41 | Sphingosine | 0.01 | 0.224 |
| 6 | 1.775 | 518.3203 | 11.09 | PC(18:3) | 0 | 0.213 |
| 7 | 1.417 | 303.2313 | 11.59 | Eicosapentaenoic Acid | 0.011 | 0.167 |
| 8 | 1.377 | 522.3548 | 11.4 | PC(18:1) | 0.014 | 0.166 |
| 9 | 1.58 | 510.3543 | 11.68 | 1-heptadecanoyl-sn-glycero-3-phosphocholine | 0.002 | 0.132 |
| 10 | 1.345 | 496.3383 | 11.09 | PC(16:0) | 0.018 | 0.117 |
| 11 | 1.454 | 225.0633 | 0.76 | Glucoheptonic acid | 0.047 | -0.027 |
| 12 | 1.65 | 164.072 | 2.1 | Phenylalanine | 0.006 | -0.091 |
| 13 | 1.372 | 249.1142 | 8.88 | Ubiquinone-1 | 0.031 | -0.096 |
| 14 | 1.527 | 118.0516 | 0.76 | Threonine | 0.013 | -0.097 |
| 15 | 1.693 | 167.0214 | 1.05 | Uric acid | 0.011 | -0.118 |
| 16 | 1.885 | 303.2341 | 13.43 | Arachidonic Acid | 0.002 | -0.121 |
| 17 | 1.424 | 281.2495 | 14.2 | Oleic Acid | 0.033 | -0.138 |
| 18 | 1.816 | 130.0874 | 1.3 | Leucine | 0.001 | -0.161 |
| 19 | 1.762 | 124.0081 | 0.76 | Taurine | 0.002 | -0.163 |
| 20 | 1.299 | 165.0545 | 1.17 | Phenylpyruvic acid | 0.025 | -0.17 |
| 21 | 1.865 | 195.0522 | 0.78 | Gluconic acid | 0.001 | -0.177 |
| 22 | 1.664 | 259.0924 | 1.14 | Ribothymidine | 0 | -0.232 |
| 23 | 1.186 | 232.1537 | 2.76 | Butyryl-L-carnitine | 0.049 | -0.241 |
| 24 | 1.469 | 182.0804 | 1.17 | Tyrosine | 0.007 | -0.245 |
| 25 | 1.551 | 118.0862 | 1.1 | Valine | 0.003 | -0.346 |
| 26 | 1.639 | 182.014 | 0.78 | Homocysteic acid | 0.005 | -0.431 |
| 27 | 1.728 | 150.058 | 1.12 | Methionine | 0 | -0.433 |
| 28 | 1.671 | 196.1 | 1.1 | n-acetyldopamine | 0 | -0.534 |
| 29 | 1.689 | 194.0841 | 1.15 | Phenylacetylglycine | 0 | -0.99 |
| 30 | 1.833 | 152.0736 | 1.32 | Phenylglycine | 0 | -1.254 |
| 31 | 1.808 | 268.1038 | 1.16 | Adenosine | 0 | -2.592 |

**Supplementary Table S4:** Differentially metabolites in PC12 cells cultured on PLLA RF after 12 h.

| NO. | VIP | mz | Rt | Metabolite | p value | Relative change (log_2_) |
| --- | --- | --- | --- | --- | --- | --- |
| 1 | 1.327 | 196.1 | 1.1 | n-acetyldopamine | 0 | 1.191 |
| 2 | 1.244 | 150.058 | 1.12 | Methionine | 0 | 0.646 |
| 3 | 1.246 | 165.0545 | 1.17 | Phenylpyruvic acid | 0 | 0.609 |
| 4 | 1.241 | 182.0804 | 1.17 | Tyrosine | 0 | 0.608 |
| 5 | 1.322 | 118.0862 | 1.1 | Valine | 0 | 0.59 |
| 6 | 1.51 | 164.072 | 2.1 | Phenylalanine | 0 | 0.526 |
| 7 | 1.225 | 300.289 | 9.41 | Sphingosine | 0 | 0.517 |
| 8 | 1.212 | 136.0756 | 1.17 | 2-Phenylacetamide | 0 | 0.489 |
| 9 | 1.114 | 466.3154 | 8.18 | Glycocholic Acid | 0.005 | 0.408 |
| 10 | 1.324 | 265.1113 | 0.76 | Thiamine | 0 | 0.404 |
| 11 | 1.368 | 271.2288 | 12.97 | hydroxypalmitic acid | 0.001 | 0.365 |
| 12 | 1.269 | 191.0203 | 1.17 | Citric acid | 0.003 | 0.352 |
| 13 | 1.096 | 156.0764 | 1.15 | Histidine | 0.008 | 0.345 |
| 14 | 1.074 | 157.0373 | 0.79 | Allantoin | 0.031 | 0.323 |
| 15 | 1.232 | 335.2185 | 10.61 | PGA2 | 0 | 0.309 |
| 16 | 1.316 | 156.0432 | 0.77 | N-Methylethanolamine phosphate | 0 | 0.304 |
| 17 | 1.241 | 189.0409 | 1.23 | 3-Dehydroquinic acid | 0.014 | 0.28 |
| 18 | 1.129 | 428.372 | 11.04 | DL-Stearoylcarnitine | 0.003 | 0.276 |
| 19 | 1.121 | 303.2313 | 11.59 | Eicosapentaenoic Acid | 0.003 | 0.276 |
| 20 | 1.203 | 454.2915 | 11.01 | Glycerophospho-N-Palmitoyl Ethanolamine | 0.001 | 0.272 |
| 21 | 1.189 | 496.3383 | 11.09 | PC(16:0) | 0.001 | 0.264 |
| 22 | 1.226 | 482.323 | 12.19 | PC(15:1) | 0 | 0.263 |
| 23 | 1.213 | 295.2261 | 10.61 | EpODE/HOTE | 0 | 0.262 |
| 24 | 1.211 | 313.2367 | 10.61 | HpODE/DiHODE | 0 | 0.259 |
| 25 | 1.223 | 192.067 | 5.26 | Phenylacetylglycine | 0.006 | 0.238 |
| 26 | 1.209 | 480.3427 | 11.43 | PC(O-16:1) | 0 | 0.237 |
| 27 | 1.125 | 510.3543 | 11.68 | 1-heptadecanoyl-sn-glycero-3-phosphocholine | 0.003 | 0.237 |
| 28 | 1.149 | 220.1176 | 2.76 | Pantothenic Acid | 0.002 | 0.236 |
| 29 | 1.164 | 522.3548 | 11.4 | PC(18:1) | 0.002 | 0.233 |
| 30 | 1.304 | 162.1122 | 0.78 | Carnitine | 0 | 0.227 |
| 31 | 1.247 | 194.0807 | 5.26 | Methylhippuric acid/Phenylacetylglycine | 0 | 0.219 |
| 32 | 1.183 | 279.2313 | 11.6 | Linolenic Acid | 0.001 | 0.214 |
| 33 | 1.002 | 130.0497 | 0.8 | Pyroglutamic acid | 0.019 | 0.212 |
| 34 | 1.526 | 440.1338 | 4.41 | Folic acid | 0.001 | 0.197 |
| 35 | 1.139 | 180.0651 | 4.85 | Hippuric acid | 0.002 | 0.195 |
| 36 | 1.085 | 206.0806 | 5.95 | Indolelactic acid | 0.007 | 0.187 |
| 37 | 1.043 | 255.2312 | 11.36 | cis-9-palmitoleic acid | 0.011 | 0.186 |
| 38 | 1.056 | 161.0958 | 11.65 | Homoglutamine | 0.007 | 0.179 |
| 39 | 1.186 | 634.4514 | 12.28 | PE(28:1) | 0.001 | 0.178 |
| 40 | 1.31 | 195.0522 | 0.78 | Gluconic acid | 0.002 | 0.171 |
| 41 | 1.191 | 167.0214 | 1.05 | Uric acid | 0.016 | 0.17 |
| 42 | 1.067 | 139.0531 | 0.8 | Nicotinamide N-oxide | 0.007 | 0.139 |
| 43 | 1.167 | 400.3416 | 10.32 | Palmitoyl-L-carnitine | 0.001 | 0.139 |
| 44 | 1.073 | 244.0998 | 2.14 | Cytidine | 0.008 | 0.129 |
| 45 | 1.1 | 205.0961 | 3.88 | Tryptophan | 0.004 | 0.108 |
| 46 | 1.173 | 170.0812 | 0.83 | Pyridoxine/Norepinephrine | 0.002 | -0.12 |
| 47 | 1.056 | 147.0763 | 0.8 | Glutamine | 0.009 | -0.155 |
| 48 | 1.153 | 259.0924 | 1.14 | Ribothymidine | 0.003 | -0.383 |
| 49 | 1.423 | 130.0874 | 1.3 | Leucine | 0.019 | -0.395 |
| 50 | 1.608 | 182.014 | 0.78 | L-Homocysteic acid | 0 | -0.494 |
| 51 | 1.153 | 853.0299 | 6.25 | Guanosine tetraphosphate adenosine | 0.002 | -0.526 |
| 52 | 1.336 | 152.0736 | 1.32 | Phenylglycine | 0 | -0.562 |
| 53 | 1.32 | 318.2998 | 8.32 | Phytosphingosine | 0 | -0.664 |
| 54 | 1.27 | 450.321 | 13.32 | Chenodeoxycholic acid glycine conjugate | 0 | -0.724 |
| 55 | 1.289 | 232.1537 | 2.76 | Butyryl-L-carnitine | 0 | -0.809 |
| 56 | 1.072 | 268.1038 | 1.16 | Adenosine | 0.01 | -1.092 |

**Supplementary Table S5:** Differentially metabolites in PC12 cells cultured on PLLA RF after 24 h.

| NO. | VIP | mz | Rt | Metabolite | p value | Relative change (log_2_) | |
| --- | --- | --- | --- | --- | --- | --- | --- |
| 1 | 1.342 | 196.1 | 1.104453 | n-acetyldopamine | 0 | 1.387 | |
| 2 | 1.292 | 156.0764 | 1.146276 | Histidine | 0 | 1.133 | |
| 3 | 1.342 | 118.0862 | 1.104617 | Valine | 0 | 1.12 | |
| 4 | 1.369 | 165.0545 | 1.173358 | Phenylpyruvic acid | 0 | 0.786 | |
| 5 | 1.365 | 136.0756 | 1.173938 | 2-Phenylacetamide | 0 | 0.72 | |
| 6 | 1.366 | 150.058 | 1.120863 | Methionine | 0 | 0.705 | |
| 7 | 1.356 | 303.2313 | 11.59077 | Eicosapentaenoic Acid | 0 | 0.539 | |
| 8 | 1.347 | 496.3383 | 11.08502 | PC(16:0) | 0 | 0.506 | |
| 9 | 1.345 | 522.3548 | 11.4007 | PC(18:1) | 0 | 0.454 | |
| 10 | 1.267 | 283.2631 | 12.09493 | Oleic Acid | 0 | 0.444 | |
| 11 | 1.344 | 510.3543 | 11.68126 | 1-heptadecanoyl-sn-glycero-3-phosphocholine | 0 | 0.433 | |
| 12 | 1.304 | 279.2313 | 11.59621 | Linolenic Acid | 0 | 0.385 | |
| 13 | 1.317 | 132.1013 | 1.309894 | Leucine | 0 | 0.345 | |
| 14 | 1.185 | 466.3154 | 8.179019 | Glycocholic Acid | 0.001 | 0.341 | |
| 15 | 1.305 | 335.2185 | 10.60763 | PGA2 | 0 | 0.341 | |
| 16 | 1.057 | 353.229 | 9.298973 | PGH2/PGE2 | 0.009 | 0.337 | |
| 17 | 1.296 | 634.4514 | 12.27505 | PE(28:1) | 0 | 0.329 | |
| 18 | 1.275 | 518.3203 | 11.08585 | PC(18:3) | 0 | 0.292 | |
| 19 | 1.276 | 295.2261 | 10.61311 | EpODE/HOTE | 0 | 0.25 | |
| 20 | 1.726 | 191.0203 | 1.17 | Citric acid | 0 | 0.239 | |
| 21 | 1.253 | 313.2367 | 10.60765 | HpODE/DiHODE | 0 | 0.209 | |
| 22 | 1.255 | 255.2322 | 13.32948 | cis-9-palmitoleic acid | 0 | 0.207 | |
| 23 | 1.26 | 194.0807 | 5.264434 | Phenylacetylglycine | 0 | 0.203 | |
| 24 | 1.142 | 265.1113 | 0.755238 | Thiamine | 0.003 | 0.2 | |
| 25 | 1.304 | 180.0651 | 4.847267 | Hippuric acid | 0 | 0.198 | |
| 26 | 1.192 | 300.289 | 9.411015 | Sphingosine | 0.002 | 0.198 | |
| 27 | 1.625 | 118.0516 | 0.76 | Threonine | 0.004 | 0.182 | |
| 28 | 1.196 | 400.3416 | 10.32258 | Palmitoyl-L-carnitine | 0.001 | 0.168 | |
| 29 | 1.691 | 164.072 | 2.1 | Phenylalanine | 0.001 | 0.166 | |
| 30 | 1.604 | 195.0522 | 0.78 | Gluconic acid | 0.005 | 0.148 | |
| 31 | 1.031 | 389.0091 | 0.667718 | dUDP | 0.018 | 0.142 | |
| 32 | 1.61 | 135.0306 | 0.8 | Hypoxanthine | 0.004 | 0.138 | |
| 33 | 1.342 | 157.0373 | 0.79 | Allantoin | 0.024 | 0.115 | |
| 34 | 1.403 | 104.036 | 0.76 | Serine | 0.019 | 0.112 | |
| 35 | 1.091 | 318.2998 | 8.321631 | Phytosphingosine | 0.006 | 0.084 | |
| 36 | 1.003 | 220.1176 | 2.763988 | Pantothenic Acid | 0.03 | 0.037 | |
| 37 | 1.239 | 182.0809 | 0.83383 | Tyrosine | 0 | -0.108 | |
| 38 | 1.028 | 170.0812 | 0.827888 | Pyridoxine/Norepinephrine | 0.015 | -0.118 | |
| 39 | 1.18 | 116.0706 | 0.791877 | Proline | 0.002 | -0.198 |  |
| 40 | 1.167 | 184.0008 | 0.599424 | Normetanephrine | 0.002 | -0.257 |  |
| 41 | 1.217 | 147.0763 | 0.801954 | Glutamine | 0.001 | -0.318 |  |
| 42 | 1.207 | 130.0497 | 0.799898 | Pyroglutamic acid | 0.001 | -0.348 |  |
| 43 | 1.313 | 148.0633 | 0.801812 | Glutamate | 0 | -0.361 |  |
| 44 | 1.361 | 152.0736 | 1.317303 | Phenylglycine | 0 | -1.713 |  |
| 45 | 1.328 | 268.1038 | 1.15729 | Adenosine | 0 | -3.43 |  |

**Supplementary Table S6:** Differentially metabolites in PC12 cells cultured on PLLA RF after 36 h.

| NO. | VIP | mz | Rt | Metabolite | p value | Relative change (log_2_) |
| --- | --- | --- | --- | --- | --- | --- |
| 1 | 1.42 | 196.1 | 1.1 | n-acetyldopamine | 0 | 1.069 |
| 2 | 1.41 | 118.0862 | 1.1 | Valine | 0 | 0.857 |
| 3 | 1.157 | 148.0961 | 1.12 | Glutamate | 0.01 | 0.845 |
| 4 | 1.359 | 156.0764 | 1.15 | Histidine | 0.001 | 0.736 |
| 5 | 1.381 | 165.0545 | 1.17 | Phenylpyruvic acid | 0 | 0.680 |
| 6 | 1.356 | 150.058 | 1.12 | Methionine | 0 | 0.635 |
| 7 | 1.376 | 136.0756 | 1.17 | 2-Phenylacetamide | 0 | 0.610 |
| 8 | 1.471 | 303.2313 | 11.59 | Eicosapentaenoic Acid | 0 | 0.536 |
| 9 | 1.419 | 132.1013 | 1.31 | Leucine | 0 | 0.502 |
| 10 | 1.441 | 255.2312 | 11.36 | cis-9-palmitoleic acid | 0 | 0.499 |
| 11 | 1.41 | 283.2631 | 12.09 | Oleic Acid | 0 | 0.498 |
| 12 | 1.364 | 454.2915 | 11.01 | Glycerophospho-N-Palmitoyl Ethanolamine | 0 | 0.434 |
| 13 | 1.333 | 335.2185 | 10.61 | PGA2 | 0.001 | 0.429 |
| 14 | 1.419 | 496.3383 | 11.09 | PC(16:0) | 0 | 0.406 |
| 15 | 1.43 | 518.3203 | 11.09 | PC(18:3) | 0 | 0.386 |
| 16 | 1.321 | 353.229 | 9.3 | PGH2/PGE2 | 0.001 | 0.375 |
| 17 | 1.409 | 522.3548 | 11.4 | PC(18:1) | 0 | 0.360 |
| 18 | 1.433 | 480.3427 | 11.43 | PC(O-16:1) | 0 | 0.356 |
| 19 | 1.399 | 161.0958 | 11.65 | Homoglutamine | 0 | 0.336 |
| 20 | 1.181 | 279.2313 | 11.6 | Linolenic Acid | 0.008 | 0.308 |
| 21 | 1.2 | 313.2367 | 10.61 | HpODE/DiHODE | 0.006 | 0.288 |
| 22 | 1.363 | 510.3543 | 11.68 | 1-heptadecanoyl-sn-glycero-3-phosphocholine | 0 | 0.277 |
| 23 | 1.133 | 295.2261 | 10.61 | EpODE/HOTE | 0.012 | 0.247 |
| 24 | 1.134 | 300.289 | 9.41 | Sphingosine | 0.012 | 0.232 |
| 25 | 1.179 | 180.0651 | 4.85 | Hippuric acid | 0.008 | 0.215 |
| 26 | 1.364 | 466.3154 | 8.18 | Glycocholic Acid | 0 | 0.144 |
| 27 | 2.091 | 164.072 | 2.1 | Phenylalanine | 0 | 0.129 |
| 28 | 1.558 | 191.0203 | 1.17 | Citric acid | 0.035 | 0.110 |
| 29 | 1.933 | 157.0373 | 0.79 | Allantoin | 0.003 | 0.045 |
| 30 | 1.673 | 498.9147 | 0.64 | D-myo-Inositol-1,3,4,6-tetraphosphate | 0.019 | -0.081 |
| 31 | 1.008 | 139.0531 | 0.8 | Nicotinamide N-oxide | 0.035 | -0.118 |
| 32 | 1.102 | 170.0812 | 0.83 | Pyridoxine/Norepinephrine | 0.018 | -0.165 |
| 33 | 1.077 | 182.0809 | 0.83 | Tyrosine | 0.02 | -0.178 |
| 34 | 1.221 | 170.011 | 0.6 | Cysteic acid | 0.005 | -0.232 |
| 35 | 1.325 | 116.0706 | 0.79 | Proline | 0.001 | -0.295 |
| 36 | 1.352 | 175.1179 | 0.66 | Arginine | 0 | -0.298 |
| 37 | 1.434 | 148.0633 | 0.8 | Glutamine | 0 | -0.394 |
| 38 | 1.448 | 130.0497 | 0.8 | Pyroglutamic acid | 0 | -0.405 |
| 39 | 1.356 | 184.094 | 0.75 | Normetanephrine | 0 | -0.488 |
| 40 | 1.479 | 152.0736 | 1.32 | Phenylglycine | 0 | -0.856 |
| 41 | 1.48 | 268.1038 | 1.16 | Adenosine | 0 | -2.253 |

**Supplementary Table S7:** Differentially mutual metabolites in PC12 cells cultured on PLLA AF for 12, 24 and 36 h.

| No. | Metabolites | AF-12h | AF-24h | AF-36h |
| --- | --- | --- | --- | --- |
| 1 | Histidine | 0.397 | 0.808 | 0.568 |
| 2 | Phenylalanine | -0.15 | -0.079 | -0.091 |
| 3 | Taurine | -0.094 | -0.071 | -0.163 |
| 4 | Valine | 0.444 | -0.406 | -0.346 |
| 5 | Leucine | 0.403 | -0.245 | -0.161 |
| 6 | Methionine | 0.38 | -0.427 | -0.433 |
| 7 | Phenylpyruvic acid | 0.319 | -0.125 | -0.17 |
| 8 | Phenylglycine | 0.131 | -1.327 | -1.254 |
| 9 | Tyrosine | -0.0075 | -0.208 | -0.245 |
| 10 | PC(18:3) | 0.139 | 0.187 | 0.213 |
| 11 | Arachidonic Acid | 0.115 | 0.16 | -0.121 |
| 12 | N-Methylethanolamine phosphate | -0.321 | 0.293 | 0.347 |
| 13 | D-Mannitol 1-phosphate | -1.222 | 0.758 | 0.591 |
| 14 | Normetanephrine | -0.409 | 0.369 | 0.351 |

**Supplementary Table S8:** Differentially mutual metabolites in PC12 cells cultured on PLLA RF for 12, 24 and 36h.

| No. | Metabolites | RF-12h | RF-24h | RF-36h |
| --- | --- | --- | --- | --- |
| 1 | Sphingosine | 0.517 | 0.198 | 0.232 |
| 2 | Eicosapentaenoic acid | 0.276 | 0.539 | 0.536 |
| 3 | PC(16:0) | 0.264 | 0.506 | 0.406 |
| 4 | EpODE/HOTE | 0.262 | 0.25 | 0.247 |
| 5 | HpODE/DiHODE | 0.259 | 0.209 | 0.288 |
| 6 | PC(18:1) | 0.233 | 0.454 | 0.360 |
| 7 | Linolenic acid | 0.214 | 0.385 | 0.308 |
| 8 | cis-9-palmitoleic acid | 0.186 | 0.207 | 0.499 |
| 9 | Methionine | 0.646 | 0.705 | 0.635 |
| 10 | Phenylpyruvic acid | 0.609 | 0.786 | 0.680 |
| 11 | Valine | 0.5895 | 1.12 | 0.857 |
| 12 | Phenylalanine | 0.526 | 0.166 | 0.129 |
| 13 | Histidine | 0.345 | 1.133 | 0.736 |
| 14 | Glutamine | -0.155 | -0.318 | -0.394 |
| 15 | Phenylglycine | -0.562 | -1.713 | -0.856 |
| 16 | Tyrosine | 0.608 | -0.108 | -0.178 |
| 17 | Pyroglutamic acid | 0.212 | -0.348 | -0.405 |
| 18 | Leucine | -0.395 | 0.345 | 0.502 |
| 19 | Adenosine | -1.092 | -3.43 | -2.253 |
| 20 | n-acetyldopamine | 1.191 | 1.387 | 1.069 |
| 21 | 2-Phenylacetamide | 0.489 | 0.72 | 0.610 |
| 22 | Glycocholic acid | 0.408 | 0.341 | 0.144 |
| 23 | Citric acid | 0.352 | 0.239 | 0.110 |
| 24 | Allantoin | 0.323 | 0.115 | 0.045 |
| 25 | PGA2 | 0.309 | 0.341 | 0.429 |
| 26 | 1-heptadecanoyl-sn-glycero-3-phosphocholine | 0.237 | 0.433 | 0.277 |
| 27 | Hippuric acid | 0.195 | 0.198 | 0.215 |
| 28 | Pyridoxine/Norepinephrine | -0.12 | -0.118 | -0.165 |

**Supplementary Table S9:** Metabolic pathways that differentially metabolites in PC12 cells cultured on PLLA AF after 12, 24 and 36 h involved in.

| No. | AF-12h | AF-24h | AF-36h |
| --- | --- | --- | --- |
| 1 | Aminoacyl-tRNA biosynthesis | Aminoacyl-tRNA biosynthesis | Aminoacyl-tRNA biosynthesis |
| 2 | Phenylalanine, tyrosine and tryptophan biosynthesis | Phenylalanine, tyrosine and tryptophan biosynthesis | Phenylalanine, tyrosine and tryptophan biosynthesis |
| 3 | Nitrogen metabolism | Nitrogen metabolism | Nitrogen metabolism |
| 4 | Phenylalanine metabolism | Phenylalanine metabolism | Phenylalanine metabolism |
| 5 | Ubiquinone and other terpenoid-quinone biosynthesis | Ubiquinone and other terpenoid-quinone biosynthesis | Ubiquinone and other terpenoid-quinone biosynthesis |
| 6 | Valine, leucine and isoleucine biosynthesis | Valine, leucine and isoleucine biosynthesis | Valine, leucine and isoleucine biosynthesis |
| 7 | Pantothenate and CoA biosynthesis | Pantothenate and CoA biosynthesis | Pantothenate and CoA biosynthesis |
| 8 | Histidine metabolism | Histidine metabolism | Histidine metabolism |
| 9 | Taurine and hypotaurine metabolism | Taurine and hypotaurine metabolism | Taurine and hypotaurine metabolism |
| 10 | Sphingolipid metabolism | Sphingolipid metabolism | Sphingolipid metabolism |
| 11 | Primary bile acid biosynthesis | Primary bile acid biosynthesis | Primary bile acid biosynthesis |
| 12 | Valine, leucine and isoleucine degradation | Valine, leucine and isoleucine degradation | Valine, leucine and isoleucine degradation |
| 13 | Cysteine and methionine metabolism | Cysteine and methionine metabolism | Cysteine and methionine metabolism |
| 14 | Tyrosine metabolism | Tyrosine metabolism | Tyrosine metabolism |
| 15 | Arachidonic acid metabolism | Arachidonic acid metabolism | Arachidonic acid metabolism |
| 16 | Purine metabolism | Purine metabolism | Purine metabolism |
| 17 | D-Glutamine and D-glutamate metabolism | D-Glutamine and D-glutamate metabolism |  |
| 18 | Arginine and proline metabolism | Arginine and proline metabolism |  |
| 19 | Alanine, aspartate and glutamate metabolism | Alanine, aspartate and glutamate metabolism |  |
| 20 | Glutathione metabolism | Glutathione metabolism |  |
| 21 | Pyrimidine metabolism | Pyrimidine metabolism |  |
| 22 | Riboflavin metabolism | Riboflavin metabolism |  |
| 23 | Fructose and mannose metabolism | Fructose and mannose metabolism |  |
| 24 | Butanoate metabolism | Butanoate metabolism |  |
| 25 | Starch and sucrose metabolism | Starch and sucrose metabolism |  |
| 26 | Galactose metabolism | Galactose metabolism |  |
| 27 | Glycolysis or Gluconeogenesis | Glycolysis or Gluconeogenesis |  |
| 28 | Porphyrin and chlorophyll metabolism | Porphyrin and chlorophyll metabolism |  |
| 29 | Amino sugar and nucleotide sugar metabolism | Amino sugar and nucleotide sugar metabolism |  |
| 30 | Biosynthesis of unsaturated fatty acids |  | Biosynthesis of unsaturated fatty acids |
| 31 | beta-Alanine metabolism |  |  |
| 32 | Glyoxylate and dicarboxylate metabolism |  |  |
| 33 | Glycosylphosphatidylinositol(GPI)-anchor biosynthesis |  |  |
| 34 | Citrate cycle (TCA cycle) |  |  |
| 35 | Glycerophospholipid metabolism |  |  |
| 36 |  | Tryptophan metabolism |  |
| 37 |  | Biosynthesis of unsaturated fatty acids |  |
| 38 |  |  | Glycine, serine and threonine metabolism |
| 39 |  |  | Fatty acid biosynthesis |

**Supplementary Table S10:** Metabolic pathways that differentially metabolites in PC12 cells cultured on PLLA RF after 12, 24 and 36 h involved in.

| No. | RF-12h | RF-24h | RF-36h |
| --- | --- | --- | --- |
| 1 | Aminoacyl-tRNA biosynthesis | Aminoacyl-tRNA biosynthesis | Aminoacyl-tRNA biosynthesis |
| 2 | Phenylalanine metabolism | Phenylalanine metabolism | Phenylalanine metabolism |
| 3 | Phenylalanine, tyrosine and tryptophan biosynthesis | Phenylalanine, tyrosine and tryptophan biosynthesis | Phenylalanine, tyrosine and tryptophan biosynthesis |
| 4 | Nitrogen metabolism | Nitrogen metabolism | Nitrogen metabolism |
| 5 | D-Glutamine and D-glutamate metabolism | D-Glutamine and D-glutamate metabolism | D-Glutamine and D-glutamate metabolism |
| 6 | Valine, leucine and isoleucine biosynthesis | Valine, leucine and isoleucine biosynthesis | Valine, leucine and isoleucine biosynthesis |
| 7 | Pantothenate and CoA biosynthesis | Pantothenate and CoA biosynthesis | Pantothenate and CoA biosynthesis |
| 8 | Sphingolipid metabolism | Sphingolipid metabolism | Sphingolipid metabolism |
| 9 | Histidine metabolism | Histidine metabolism | Histidine metabolism |
| 10 | Ubiquinone and other terpenoid-quinone biosynthesis | Ubiquinone and other terpenoid-quinone biosynthesis | Ubiquinone and other terpenoid-quinone biosynthesis |
| 11 | Biosynthesis of unsaturated fatty acids | Biosynthesis of unsaturated fatty acids | Biosynthesis of unsaturated fatty acids |
| 12 | Alanine, aspartate and glutamate metabolism | Alanine, aspartate and glutamate metabolism | Alanine, aspartate and glutamate metabolism |
| 13 | Cysteine and methionine metabolism | Cysteine and methionine metabolism | Cysteine and methionine metabolism |
| 14 | alpha-Linolenic acid metabolism | alpha-Linolenic acid metabolism | alpha-Linolenic acid metabolism |
| 15 | Purine metabolism | Purine metabolism | Purine metabolism |
| 16 | Valine, leucine and isoleucine degradation | Valine, leucine and isoleucine degradation | Valine, leucine and isoleucine degradation |
| 17 | Pyrimidine metabolism | Pyrimidine metabolism | Pyrimidine metabolism |
| 18 | Tyrosine metabolism | Tyrosine metabolism | Tyrosine metabolism |
| 19 | Glyoxylate and dicarboxylate metabolism | Glyoxylate and dicarboxylate metabolism | Glyoxylate and dicarboxylate metabolism |
| 20 | Citrate cycle (TCA cycle) | Citrate cycle (TCA cycle) | Citrate cycle (TCA cycle) |
| 21 | Primary bile acid biosynthesis | Primary bile acid biosynthesis | Primary bile acid biosynthesis |
| 22 | Thiamine metabolism | Thiamine metabolism |  |
| 23 | Glycosylphosphatidylinositol (GPI)-anchor biosynthesis | Glycosylphosphatidylinositol (GPI)-anchor biosynthesis |  |
| 24 | Glycerophospholipid metabolism | Glycerophospholipid metabolism |  |
| 25 | Fatty acid metabolism | Fatty acid metabolism |  |
| 26 | Arginine and proline metabolism |  | Arginine and proline metabolism |
| 27 |  | Glutathione metabolism | Glutathione metabolism |
| 28 |  | Butanoate metabolism | Butanoate metabolism |
| 29 |  | Porphyrin and chlorophyll metabolism | Porphyrin and chlorophyll metabolism |
| 30 |  | Fatty acid biosynthesis | Fatty acid biosynthesis |
| 31 | One carbon pool by folate |  |  |
| 32 | Folate biosynthesis |  |  |
| 33 | Tryptophan metabolism |  |  |
| 34 | Glutathione metabolism |  |  |
| 35 |  | Arginine and proline metabolism |  |
| 36 |  | Cyanoamino acid metabolism |  |
| 37 |  | Methane metabolism |  |
| 38 |  | Glycine, serine and threonine metabolism |  |
| 39 |  |  | Taurine and hypotaurine metabolism |
| 40 |  |  | Inositol phosphate metabolism |

**Supplementary Table S11:** Important metabolic pathways and pathway impact in PLLA AF groups.

| No. | Metabolic pathways | AF-12h | AF-24h | AF-36h |
| --- | --- | --- | --- | --- |
| 1 | Phenylalanine, tyrosine, and tryptophan biosynthesis | 1 | 1 | 1 |
| 2 | Valine, leucine and isoleucine biosynthesis | 0.66666 | 0.66666 | 0.66666 |
| 3 | Phenylalanine metabolism | 0.64815 | 0.64815 | 0.64815 |
| 4 | Taurine and hypotaurine metabolism | 0.42857 | 0.42857 | 0.42857 |
| 5 | Arachidonic acid metabolism | 0.32601 | 0.32601 | 0.32601 |
| 6 | Histidine metabolism | 0.24194 | 0.24194 | 0.24194 |
| 7 | Aminoacyl-tRNA biosynthesis | 0 | 0 | 0 |
| 8 | D-glutamine and D-glutamate metabolism | 1 | 1 |  |
| 9 | Alanine, aspartate and glutamate metabolism | 0.40928 | 0.40928 |  |
| 10 | Arginine and proline metabolism | 0.23975 | 0.23975 |  |
| 11 | Glyoxylate and dicarboxylate metabolism | 0.2963 |  |  |
| 12 | Tyrosine metabolism |  | 0.29436 |  |

**Supplementary Table S12:** Important metabolic pathways and pathway impact in PLLA RF groups.

| No. | Metabolic pathways | RF-12h | RF-24h | RF-36h |
| --- | --- | --- | --- | --- |
| 1 | Phenylalanine, tyrosine and tryptophan biosynthesis | 1 | 1 | 1 |
| 2 | alpha-Linolenic acid metabolism | 1 | 1 | 1 |
| 3 | Valine, leucine and isoleucine biosynthesis | 0.66666 | 0.66666 | 0.66666 |
| 4 | Phenylalanine metabolism | 0.64815 | 0.64815 | 0.64815 |
| 5 | Glyoxylate and dicarboxylate metabolism | 0.2963 | 0.2963 | 0.2963 |
| 6 | Histidine metabolism | 0.24194 | 0.24194 | 0.24194 |
| 7 | Aminoacyl-tRNA biosynthesis | 0 | 0 | 0 |
| 8 | Thiamine metabolism | 0.4 | 0.4 |  |
| 9 | D-Glutamine and D-glutamate metabolism |  | 1 | 1 |
| 10 | Alanine, aspartate and glutamate metabolism |  | 0.40928 | 0.40928 |
| 11 | Methane metabolism |  | 0.4 |  |
| 12 | Glycine, serine and threonine metabolism |  | 0.2428 |  |
| 13 | Arginine and proline metabolism |  |  | 0.23975 |

(a)

(b)

**Supplementary Figure S1:** The metabolite variability achieved by PCA in both positive (a) and negative (b) ion modes.

(a)

(b)

**Supplementary Figure S2:** The relative concentration change of phenylalanine (a) and tyrosine (b).
